# DFT studies on the structural and vibrational properties of polyenes

**DOI:** 10.1007/s00894-016-2969-1

**Published:** 2016-04-06

**Authors:** Teobald Kupka, Aneta Buczek, Małgorzata A. Broda, Michał Stachów, Przemysław Tarnowski

**Affiliations:** Faculty of Chemistry, University of Opole, 48 Oleska Street, 45-052 Opole, Poland

**Keywords:** C = C bond, C-C bond, All-trans polyenes, All-cis polyenes, IR and Raman spectroscopy, DFT

## Abstract

Detailed density functional theory (DFT) calculations on the structure and harmonic frequencies of model all-trans and all-cis polyenes were undertaken. For the first time, we report on the convergence of selected B3LYP/6-311++G** and BLYP/6-311++G** calculated structural parameters resulting from a systematic increase in polyene size (chains containing 2 to 14 C = C units). The limiting values of the structural parameters for very long chains were estimated using simple three-parameter empirical formulae. BLYP/6-311++G** calculated ν(C = C) and ν(C–C) frequencies for all-trans and all-cis polyenes containing up to 14 carbon–carbon double bonds were used to estimate these values for very long chains. Correction of raw, unscaled vibrational data was performed by comparing theoretical and experimental wavenumbers for polyenes chains containing 3 to 12 conjugated C = C units with both ends substituted by tert-butyl groups. The corrected ν(C = C) and ν(C–C) wavenumbers for all-trans molecules were used to estimate the presence of 9 – 12 C = C units in all-trans polyene pigment in red coral.

**Electronic supplementary material:**

The online version of this article (doi:10.1007/s00894-016-2969-1) contains supplementary material, which is available to authorized users.

**Graphical abstract:**
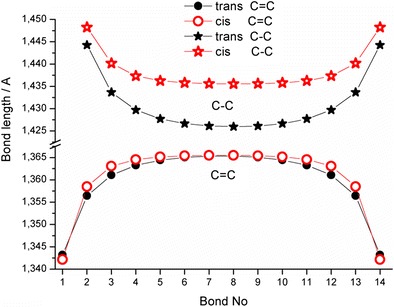
Detailed density functional theory (DFT) calculations on the structure and harmonic frequencies of model all-trans and all-cis polyenes were undertaken. For the first time, we report on the convergence of selected B3LYP/6-311++G** and BLYP/6-311++G** calculated structural parameters resulting from a systematic increase in polyene size (chains containing 2 to 14 C=C units). The limiting values of the structural parameters for very long chains were estimated using simple three-parameter empirical formulae.

## Introduction

**Scheme 1:**
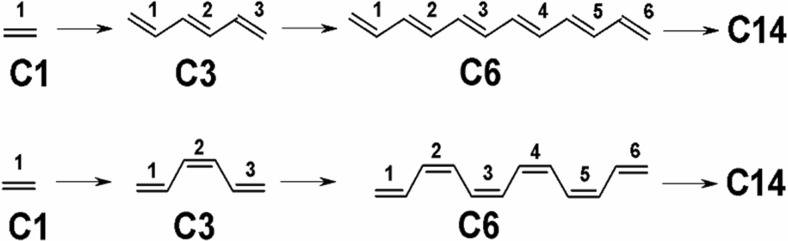
Ethylene and model all-trans (*top*) and all-cis (*bottom*) polyenes containing 2–14 conjugated C = C units

Ethylene, and all-trans linear polyenes containing two or more conjugated double bonds, form a family of unsaturated hydrocarbons present in nature [1–11]. The all-cis homolog series are formed by chains containing three or more C = C units and are thermodynamically unstable. Ethylene and the first members of all-trans and all-cis polyenes, containing up to 14 conjugated double bonds, are shown schematically in Scheme 1. The double carbon–carbon bonds are numbered systematically along the chain. We arbitrary call them C1–C14 compounds (see also [12]). Addition of consecutive units formally produces systems of infinite length (C∞). These compounds are one dimensional (1D) molecular rods, highly symmetrical, and practically nonpolar.

The presence of conjugated C = C bonds in polyenes leads to a partial delocalization of π-electrons. The degree of electron delocalization in such systems containing 1 to 4 C = C bonds was studied experimentally and theoretically by Craig and coworkers [13]. They calculated the length of a “naked C–C bond” in a twisted form of butadiene, e.g., a single carbon–carbon bond formed by two sp^2^ carbon atoms not influenced by π-electron overlapping. Thus, in conjugated molecules, an increase of C = C and shortening of the adjacent C–C bond occurs. This effect should be significantly more pronounced for longer polyene chains. Obviously, the presence of trans- or cis- isomers have a large impact on the degree of electron delocalization [6, 10, 11, 14–16]. As a result, all-trans polyenes are significantly better electrical current conductors than their all-cis counterparts [11, 15, 17].

Both the unsaturated chain ends and single hydrogen atoms along the hydrocarbon chain are often replaced by larger substituents (for example, methyl groups and small rings are present in β-carotene). In fact, β-carotene (see [18] for an example of a thorough theoretical study on vibrations of all-trans and all-cis forms of β-carotene) and/or all-trans polyenes containing 8–16 C = C units have been considered as primary constituents of pigments in plants, precious pink and red coral, as well as mollusks and parrots’ red feathers [19].

Conjugated C = C bonds are also essential structural fragments of numerous biological systems that are sensitive to light. Simplified models of such derivatives are used in both experimental and theoretical studies. For example, high level theoretical studies on the structure and photochemical properties of shorter trans- and cis-polyene fragments, including structurally modified 11-cis-retinal chromophores (as retinal protonated Schiff bases), were reported recently by Andruniow and coworkers [20]. Besides, the bond length alternation (BLA calculated as difference between C–C and C = C bond lengths) in conjugated systems was also studied recently using double hybrid density functionals [21] as well as higher levels of theory [10, 20, 22, 23]. In particular, the BLA index is very important for studies of conducting polymers, including polyenes (or polyacetylenes), and is also related to the HOMO/LUMO energy gap. Due to difficulties in reliable prediction of BLA using Hartree-Fock or DFT methodology, many performance tests have been reported [10, 16, 23–26]. These studies tested a wide selection of density functionals and MP2 methods, and the results were compared to the “gold standard”: coupled cluster with single, double and perturbationally included triple excitations, CCSD(T). Unfortunately, the latter method can be applied only to the first members of the polyene series.

The selection of systematically changing molecular models is advantageous from a theoretical point of view [27]. In such cases, the trend and continuous changes of structural, energetic and spectroscopic parameters are easier to predict and explain than absolute values [27]. This also could result in the cancellation of some systematic errors, and the accuracy of such calculations is not so critical. For these reasons, the use of density functional theory (DFT) [28–30], as a practical compromise between accuracy and calculation speed, is a good choice for studying larger molecules of biological interest.

In our previous DFT study, we noticed and analyzed a systematic convergence of C–C bonds and changes in π-electron delocalization in benzene rings upon addition of subsequent 1–8 rings and formation of one-dimensional linear acenes [27]. Apart from structural parameters, we noticed regular changes in the HOMO and LUMO values in these systems, as well as a decrease in selected Raman active harmonic frequencies in systematically enlarged cyclacenes [27].

To the best of our knowledge, only limited HF, MP2 and DFT calculations of systematic changes in stretch C = C vibrations in C1–C9 [31], as well as C1–C16 [12] all-trans polyenes have been reported [32, 33]. Furthermore, the characteristic Raman features of all-trans and all-cis polyenes have not been analyzed in detail.

It seems worth analyzing not only the Raman active stretch C = C band but also the second intense signal (stretch C–C) in all-trans and all-cis polyenes [3]. This approach could produce additional, and to some extent, complementary data that could better explain the observed spectral features and the nature of longer polyenes [3]. Obviously, such investigations could benefit not only environmental studies related to red corals, including global warming and greenhouse effects, but also the preparation and production of new components in material science, related to better interaction of organic pigments with light and the production of electrical current conductors and semiconductors [15].

The aim of this study was to correlate theoretically predicted selected structural and vibrational parameters of model all-trans and all-cis polyenes using DFT methodology with BLYP [34–36] and B3LYP [29, 34, 37] functionals with the position of two characteristic bands observed in the Raman reflectance spectra of red coral [12]. Hopefully, the combined experimental and theoretical studies will shed more light on the controversial nature of coral pigment.

The B3LYP density functional was selected as the most popular one, producing generally good energy, structure and spectroscopic properties; BLYP was also recently shown to reproduce well the frequencies of ethylene and its fluorinated derivatives [38, 39].

However, the challenging problem of accuracy [16] in describing BLA in conjugated systems, photochemical properties or static and dynamic electron correlation in analyzing π-conjugation in the ground and excited states will not be discussed further here (see recent work on 1,3-butadiene [4]).

## Methods

**Scheme 2:**
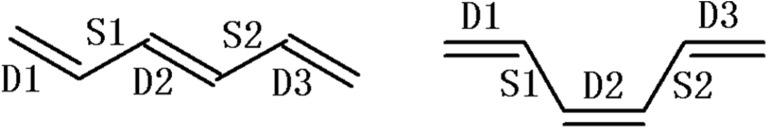
Numbering pattern of double (Dn) and single (Sn) bonds in all-trans (*left*) and all-cis (*right*) polyene molecules C3 containing three double bonds

Ethylene and C2–C14 all-trans and all-cis polyenes with chain ends capped by hydrogen atoms were selected as model compounds (see Scheme 1). Starting from the left chain end, all double (Dn) and single (Sn) bonds, where *n* = 1–14, were labeled consecutively. As an example, the bond labeling pattern for trans- and cis-C3 isomers is illustrated in Scheme 2.

Experimental studies on C3–C12 systems, with both chain ends capped by tert-butyl groups have been reported [3]. In the current study, this family of homology compounds was selected and modeled for direct verification of the accuracy of our theoretical vibrational frequencies with respect to reported experimental harmonic frequencies for these end-substituted polyenes [3], and only C = C and C-C bond lengths and the corresponding stretch frequencies were analyzed.

Unconstrained geometry optimization of all studied polyene compounds at BLYP/6-311++G** and B3LYP/6-311++G** levels of theory was performed in the Gaussian 09 program [40], selecting tight convergence criteria and followed by subsequent vibrational analysis. The latter calculations allowed prediction of both IR and Raman spectra by selecting the following keyword option: Freq = Raman. The optimized geometries were true energy minima on the potential energy surface since no imaginary frequencies were obtained as a result of frequency calculations. In an initial test on ethylene, anharmonic frequencies were calculated using the second order vibrational perturbation theory (VPT2) developed by Barone and coworkers [41, 42] and applied in the recent version D.01 of Gaussian 09 [40]. Moreover, ethane was selected as a reference molecule having a typical C–C bond (see also [16]), and its structure was optimized at the same level of theory.

In analogy to the estimation of energy in the complete basis set (CBS) limit, the structural and spectroscopic parameter Y(n) changes upon increasing chain length (*n* = 1,2,3,… ∞, where *n* is the number of C = C units) were estimated toward infinity using an exponential-type three-parameter function [43, 44]:

1 $$ Y\left(\mathrm{n}\right)=Y\left(\infty \right)+A \exp \left(-n/B\right) $$

In this formula, A and B are fitting parameters and Y(∞) is the estimated value of C = C, C–C bond lengths or ν(C = C) and ν(C–C) frequencies for polyene systems containing “*n* = ∞” C = C units.

## Results and discussion

### Selected structural parameters of all-trans and all-cis polyenes

**Fig. 1:**
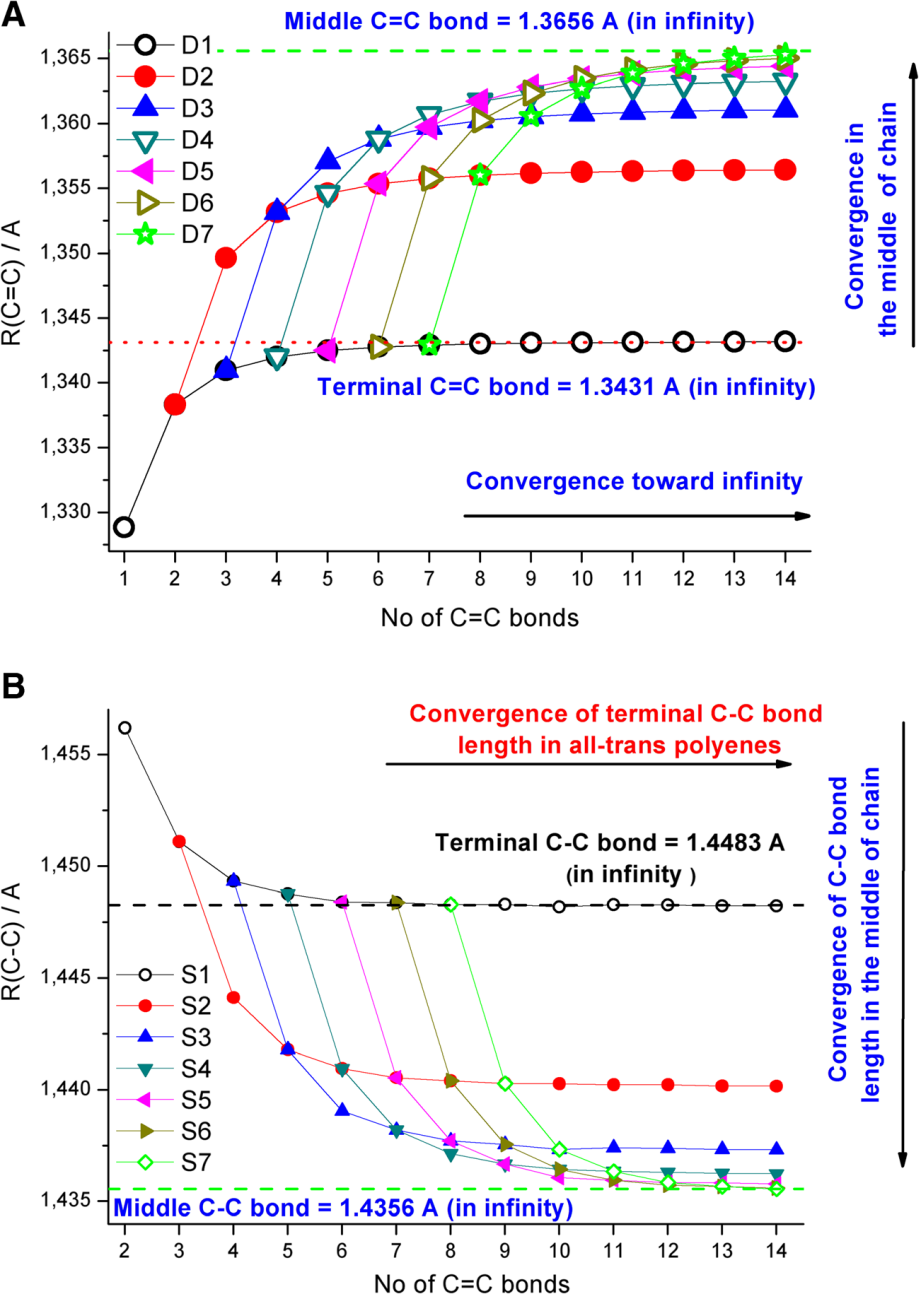
Changes in B3LYP/6-311++G**-calculated C = C (**a**) and C–C (**b**) bond lengths in all-trans polyenes with 1–14 conjugated double bond units. Convergences of terminal C = C and C–C bond lengths in the middle of molecule with increasing chain length are indicated

Force constants and vibrational stretching frequencies in polyenes are directly related to their C = C and C–C bond lengths [9, 45]. For this reason, we first analyzed in detail the patterns of B3LYP/6-311++G**-calculated C = C and C–C bond length changes upon increasing the length (*n*) of all-trans polyenes (Fig. 1). For brevity, all the calculated C = C and C–C bond lengths in all-trans C1–C14 molecules are gathered in Tables S1A and S1B in the supplementary material.

The change in terminal C = C bond length as a result of increasing the all-trans polyene chain length, expressed by the number of double bonds (*n*) in C1–C14 molecules is shown graphically in Fig. 1a (see also Table S1A in the supplementary material). Very regular patterns of change (initial continuous increase leading to a saturation plateau at 1.3431 Å for molecules larger than C7) suggest a convergence of terminal double bond length upon increasing the (*n*) value toward infinity (see also [12] and [27]). In addition, a second convergence path (and increase of length to the limiting value of 1.3656 Å) of double bonds located in the middle of chain (here shown for seven C = C bonds) is also apparent.

As expected, due to the increased importance of electron delocalization, the B3LYP/6-311++G**-calculated single C–C bond becomes shorter upon increasing the all-trans polyene chain length (see Fig. 1b and Table S1B in the supplementary material). Thus, in the case of the carbon–carbon single bond, the opposite effect to that observed with C = C bonds upon chain length increase is seen.

The observed increasing (or decreasing) exponential-like patterns of C = C and C–C bond length changes in all-trans polyenes upon systematic increase of the molecular system size is reminiscent of the structural dependencies calculated for linear acenes, closed rings or “belts” of cyclacenes and model single-walled carbon nanotubes [9, 27]. In fact, similar to systems containing linearly conjugated benzene rings [27], polyenes show strong electron delocalization, and a saturation effect is also apparent for oligomers containing more than 6–7 C = C units. This is manifested in their structural and vibrational changes, which are dependent on chain length [12].

**Fig. 2a–d:**
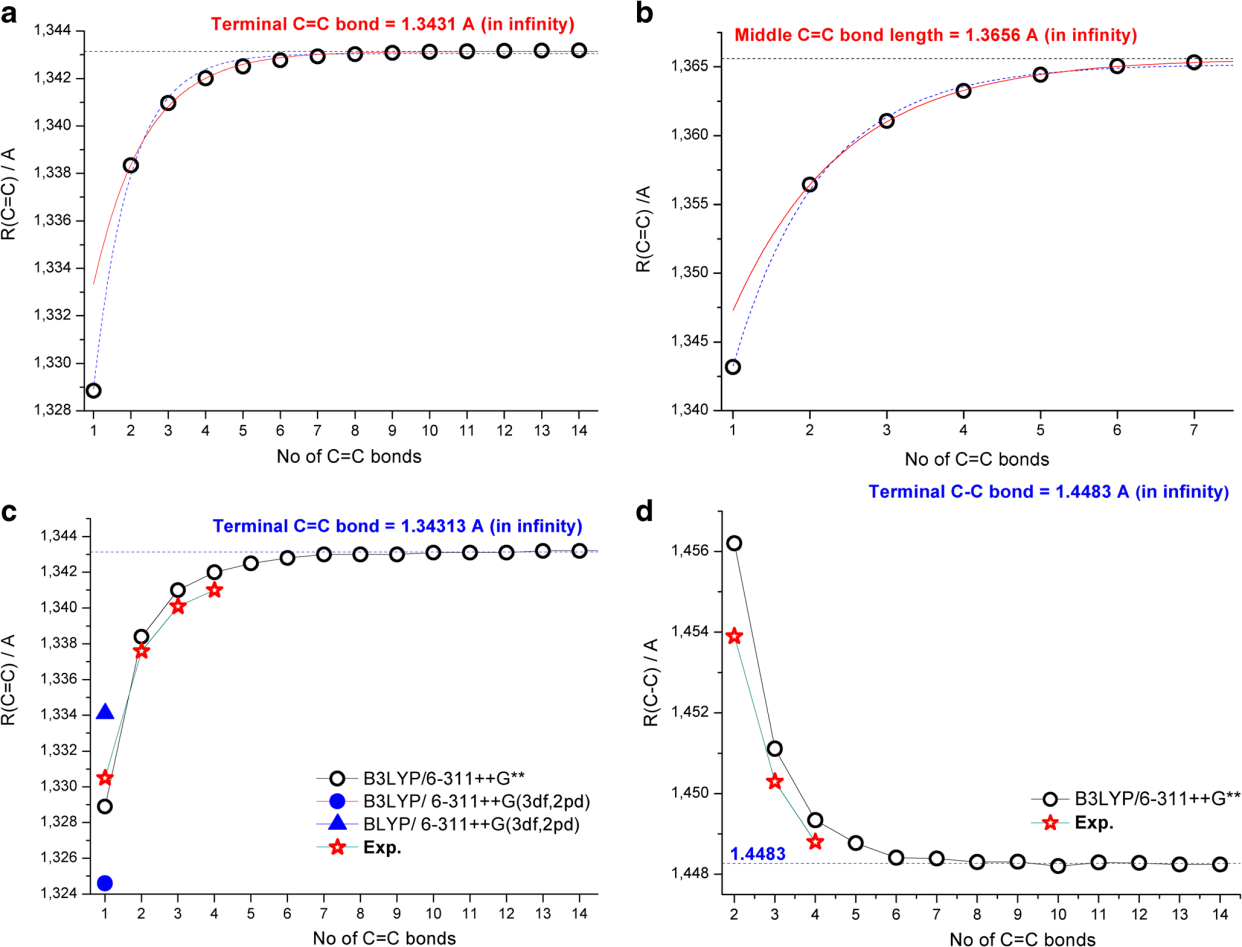
Convergence of B3LYP/6-311++G**-calculated C = C and C–C bond lengths in all-trans polyenes. **a** Result of three parameter fit using 1–14 (*dotted blue line*) and 2–14 terminal C = C bonds (*red continuous line*). **b** Result of three parameter fit using 1–7 (*dotted blue line*) and 2–14 (*red continuous line*) C = C bonds in the middle of the chain. **c** Comparison of available experimental and high level theoretical results for terminal C = C bonds in ethylene and small all-trans polyenes with B3LYP/6-311++G** results. **d** Comparison of available experimental and high level theoretical results for terminal C–C bonds in ethylene and small all-trans polyenes with B3LYP/6-311++G** results

In order to explain the convergence patterns shown in Fig. 1, we analyzed in detail the trends in C = C and C–C bond length changes in all-trans C1–C14 molecules. The fitting details allowing estimation of characteristic C = C bond lengths for very long all-trans polyenes (or infinity) with the help of Eq. (1) are gathered in Fig. 2. The convergence of terminal C = C bond length toward infinite chain length is nicely fitted using *n* = 2 – 14 (Fig. 2a). Inclusion of ethylene (*n* = 1, represented by a dotted line) produces a somewhat worse fit but the limiting value is the same (1.3431 Å). A similar convergence of the middle C = C unit toward the value of 1.3656 Å is observed in Fig. 2b (fit using 2 – 7 points is better than starting from ethylene). To show the accuracy of B3LYP/6-311++G**-calculated C = C bond lengths of ethylene and the terminal unit in all-trans polyenes, Fig. 2c compares the available experimental and high level theoretical data [1, 4, 5, 46] with the values obtained in this study.

The ethylene C = C bond length calculated with B3LYP and BLYP density functionals combined with a larger basis set [6-311++G(3df,2pd)] is also shown. It is apparent from Fig. 2c that the pattern of C = C bond length changes upon increasing the chain length is very accurately predicted at the B3LYP/6-311++G** level of theory, and only a small overestimation of about 0.001 Å is visible. Elongation of all-trans chain results in regular shortening of the terminal C–C bond length (Fig. 2d). Also in this case, theory overestimates the available experimental and high-level theoretical results for C1 (or C2)–C4 molecules from the literature [1, 4, 5, 16, 46] by less than 0.001 Å. Thus, our DFT calculated C = C and C–C bond lengths for ethylene and shorter all-trans polyenes show similar trends (and are fairly close) to accurate CCSD(T) results reported earlier [1, 4, 5, 16, 46].

For completeness, the details of fitting terminal C = C and C–C bond lengths in the middle of all-trans and all-cis polyene chains, calculated with B3LYP and BLYP density functionals combined with the 6-311++G** basis set, are gathered in Figs. S1–S5 and Tables S1–S2 in the supplementary material. In short, in every case, the changes of DFT calculated bond lengths vs (*n*) are also regular, and it is possible to use an exponential fitting to estimate the limiting value of terminal C = C and C–C bond lengths, as well as in the middle of the chains.

**Fig. 3:**
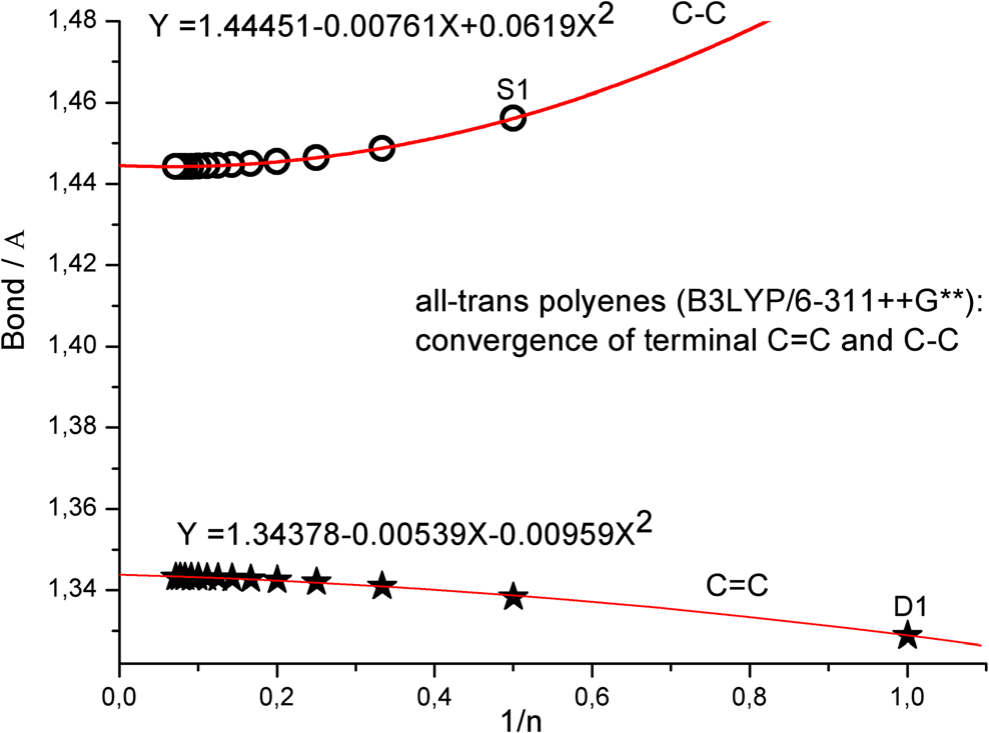
An alternative way of presenting terminal C = C and C–C bond length convergence in ethylene and all-trans C2-C14 polyenes vs chain length (as function of 1/*n*, see Fig. 1 in [47]). Polynomial instead of linear fit was used

The method of graphical presentation of our results in Figs. 1 and 2 and in the following figures needs some additional comment (for additional studies on fitting of polyene structural parameters see also [10], [23], and [25]). In 1999, Salvi and coworkers [47] reported on B3LYP/6-31G*-predicted structure and vibrational spectra of C2–C9 polyenes and plotted the C = C and C–C bonds vs. 1/*n*. They used shorter polyene chains (*n* = 2–9) and excluded ethylene from the fits (see Fig. 1 in [47]). To compare our data estimated for very long polyene chains with their results, in Fig. 3 we plotted the terminal C = C and C–C bond lengths, calculated at B3LYP/6-311++G** level of theory for all-trans C1–C14 molecules vs. 1/*n*. Interestingly, our nonlinear (second order polynomial) fits in Fig. 3 produced the same values Y(∞) for very long chains. Hence, the use of linear fitting to such sets of results seems to be less efficient than nonlinear fit. Anyhow, the two methods of graphical presentation seem to be equivalent, and to produce the same Y(∞), but we prefer our approach, which directly and nicely shows the convergence pattern upon increasing the chain length. Besides, it is worth mentioning that, some time ago, Furukawa [9] reported on successful fitting of polyene infrared frequencies using an exponential-like mathematical expression similar to Eq. (1).

**Fig. 4:**
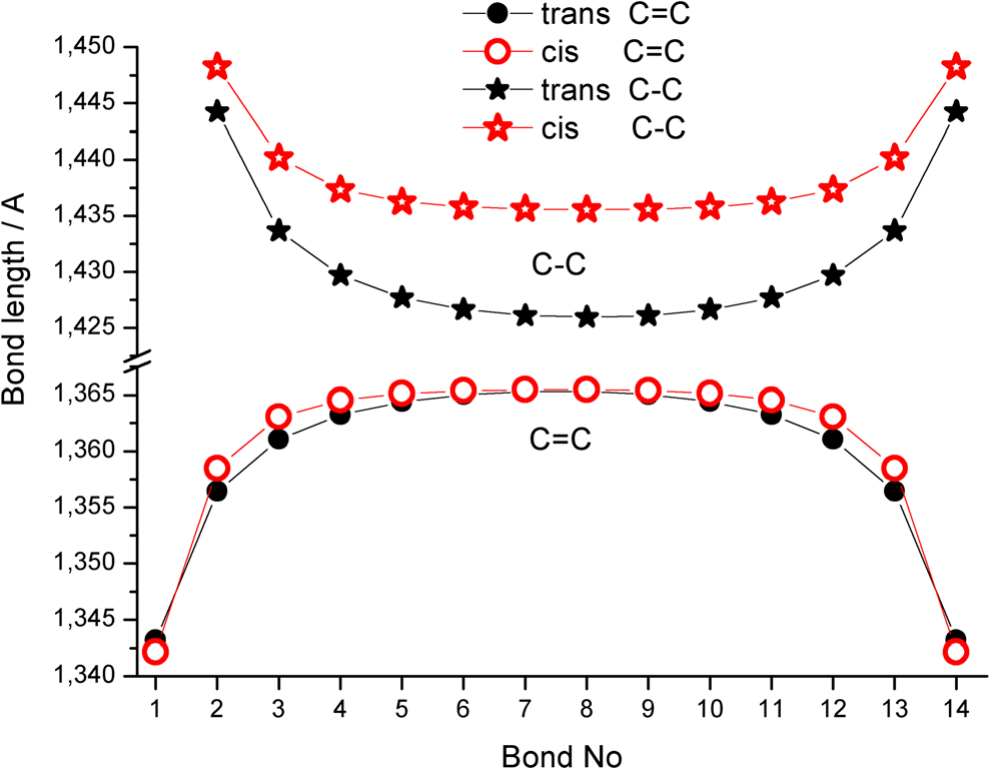
Opposite patterns of terminal C–C and C = C bond lengths change along all-trans and all-cis polyene chains containing 14 conjugated double bonds (B3LYP/6-311++G** results)

The opposite behavior of B3LYP/6-311++G** calculated C = C and C–C bond lengths along polyene chains containing 14 C = C bonds is clearly visible in Fig. 4. There is a characteristic difference between C–C bonds in all-trans and all-cis polyenes: a significantly larger deformation (shortening) of length upon increasing the chain dimension is observed for all-trans systems. The opposite effect is observed in all-cis polyenes: a significantly smaller deformation of structure, probably due to less efficient electron delocalization (Fig. 4). This observation nicely explains the significantly higher electrical current conduction in all-trans polyenes [7, 17].

A general picture showing the limiting values of terminal C–C and C = C bond lengths and in the middle of all-trans and all-cis polyenes for *n* = ∞, as well as for ethylene, and the performance of both density functionals is apparent from Table 1. The shrinkage of polyenes C–C and lengthening of C = C bonds is also demonstrated by comparison of the estimated values with “isolated” bonds in reference molecules—ethane, ethylene, and the so-called “naked” C-C sp^2^-sp^2^ bond [13]. It is apparent from Table 1 that the relative changes in bond lengths predicted with BLYP and B3LYP density functionals are similar. Besides, more significant changes upon increasing the length of the polyene chain are observed for C = C and C–C bonds in the middle of the structure than at the end of the studied molecules. For example, the B3LYP/6-311++G** calculated lengthening of the C = C bond at the end (terminal unit) and in the middle of the molecule in very long all-trans polyenes are about 0.014 and 0.037 Å, respectively. However, the corresponding changes in terminal C–H bond, and the CCH angle at the chain end are almost negligible (not shown). Thus, the two terminal C–H bonds in ethylene (1.0850 Å) vary only slightly in the C14 molecule (1.0854 and 1.0831 Å, on average 1.0842 Å). Similarly, the C–H bond in the middle of the C2 molecule (1.0882 Å) increases to 1.0889 Å in the C14 molecule. The terminal CCH angle is also very insensitive to the size of polyene (121.74° in ethylene and 121.53° in C14).

**Table 1:** Estimated C = C and C–C bond lengths for very long all-trans and all-cis polyene chains (from three-parameter fitting of B3LYP and BLYP values calculated with 6-311++G** basis set) and deviations from the reference C = C and C–C values

Bond	All-trans		All-cis	
	B3LYP	BLYP	B3LYP	BLYP
C = C				
Terminal	1.343	1.357	1.342	1.355
Middle	1.366	1.387	1.365	1.385
C-C				
Terminal	1.444	1.446	1.448	1.450
Middle	1.426	1.422	1.436	1.433
Reference^a^				
C = C in C_2_H_4_	1.329	1.338	1.329	1.338
“naked” C-C sp^2^-sp^2^	1.482^b^			
C-C in C_2_H_6_	1.531	1.542	1.531	1.542
Deviation				
C = C from C_2_H_4_				
Terminal	0.014	0.018	0.013	0.017
Middle	0.037	0.048	0.037	0.046
C-C from “naked” C-C				
Terminal	−0.037	−0.036	−0.034	−0.032
Middle	−0.056	−0.060	−0.046	−0.048

^a^This work

^b^from [13]

### BLYP/6-311++G** calculated ν(C = C) and ν(C–C) vibrations of all-trans and all-cis polyenes

In our recent studies on ethylene [39] and its fluorinated derivatives [38], we observed significantly more accurate prediction of experimental frequencies using BLYP rather than B3LYP density functional for calculations of harmonic vibrations. Obviously, the anharmonic B3LYP frequencies of ethylene better reproduce experimental data but are too expensive computationally for longer polyenes. Thus, as a practical compromise between accuracy and calculation expense, in the current study we will only discuss the BLYP/6-311++G**-calculated frequencies using a harmonic model. At this point, we would like to mention shortening of cpu timing as another positive aspect of calculating Raman frequencies in the current study using pure BLYP density functional instead of hybrid B3LYP functional. Thus, following the suggestion of a reviewer, the Raman frequencies were calculated on 24 processors and 59 GB memory for a previously optimized all-trans C10 molecule (or C_20_H_22_ molecule with 594 basis set functions) with both density functionals and the 6-311++G** basis set. There was a cpu saving of about three times using BLYP/6-311++G** calculations, as compared to the B3LYP result (7.5 vs. 20.5 h).

**Fig. 5:**
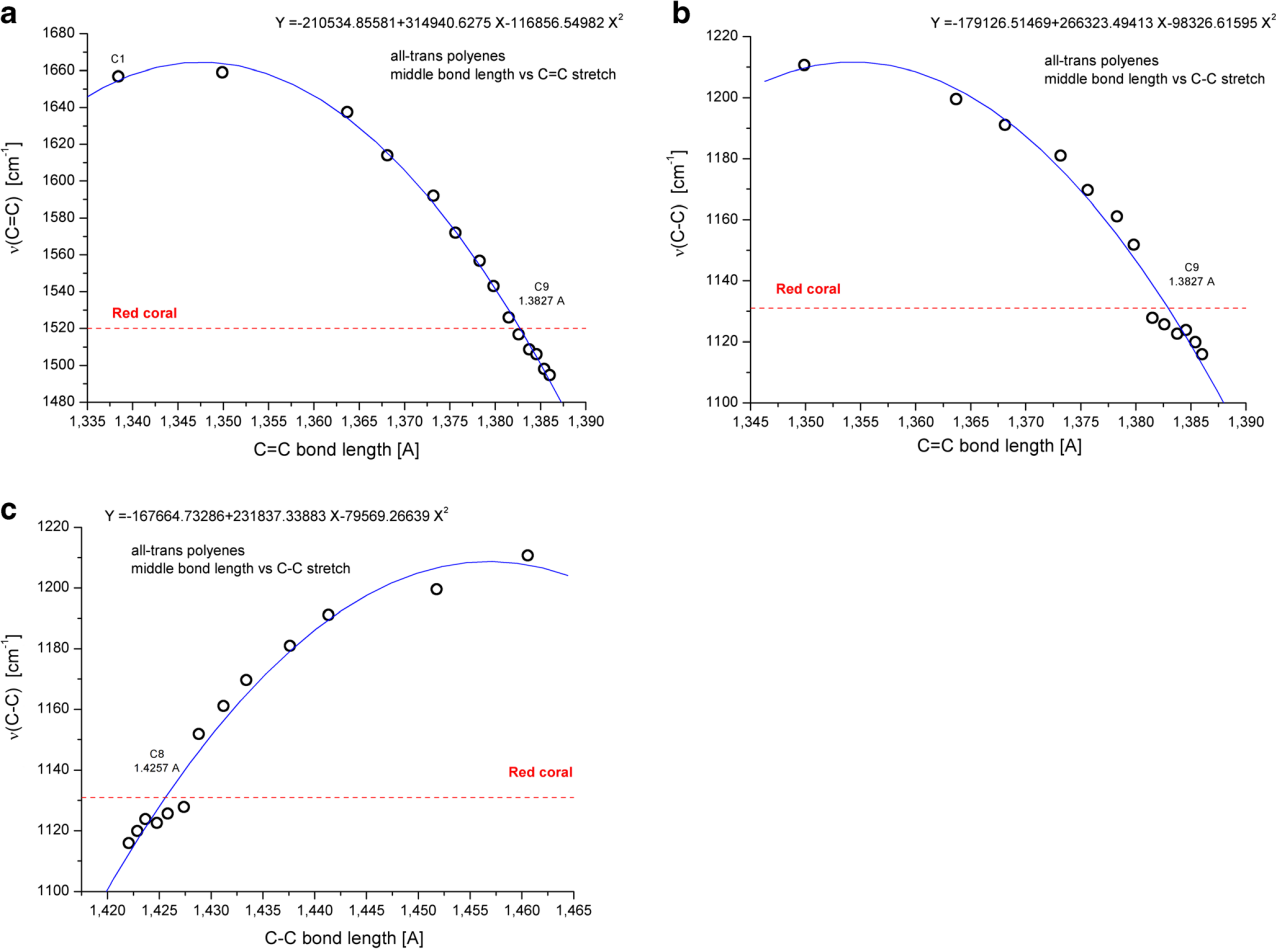
Dependence of **a** νC = C vs the length of C = C bond, **b** νC-C vs the length of C = C bond, and **c** νC–C vs the length of C–C bond in the middle of C2–C14 all-trans polyene chains (the C1 result for ethylene is also included). The trend line obtained from second order polynomial fit and experimental Raman frequency for red coral is also shown

The BLYP/6-311++G**calculated IR spectra of polyenes (not shown) are significantly more complex and crowded than the corresponding Raman spectra. The latter contain only two strong and well separated peaks due to ν(C = C) and ν(C–C) vibrations. The other peaks are of significantly lower intensity. The positions of these two bands (raw, unscaled values) change in a nonlinear and nonuniform way upon increasing the chain length (see also [48]), as a result of the corresponding modifications of C = C and C–C bond lengths. This is apparent from Fig. 5a–c with trend lines obtained from second order polynomial fits. These graphs also indicate the possible number of C = C units in the studied samples of red coral. In addition, it is possible to get a rough estimate of C = C and C–C bond lengths in the pigment by supporting the assignment of recent experimental data (see our earlier study [12]) with the results of simplified molecular modeling, and performing calculations for isolated polyene molecules without solvent or solid matrix effects [12].

As demonstrated earlier in Figs. 2 and 4, a significant degree of deformation of typical double C = C and single C–C bond lengths was predicted theoretically for both families of all-trans and all-cis polyenes (see also [21]). These changes are not linear, and saturate from the end of chain toward the middle of molecule for C7 and longer chains. Similar observations were recently reported for linear acenes [27]. Thus, such changes should be also reflected in the calculated most intensive Raman active C = C and C–C vibrations for the studied molecular series (Table 2). Moreover, the corresponding harmonic frequencies tend to decrease for some lengths of polyene chain as a function of Cn and saturate for longer molecules. This is illustrated in Fig. 5, with the added lines indicating positions of the reported Raman bands in red coral [12].

**Table 2:** BLYP/6-311++G**-calculated harmonic frequencies of ν(C = C) and ν(C–C) vibrations in ethylene, and first members of all-trans and all-cis Cn polyenes containing up to 14 conjugated carbon–carbon double bonds. The most intense Raman active modes are shown^a^

C_n_	ν(C = C)		ν(C–C)	
	trans	cis	trans	Cis
1	1628.93	1628.93		
2	1629.93	1629.93	1193.86	1193.86
3	1607.24	1605.9	1185.64	1253.31
4	1582.56	1573.59	1180.06	1260.07
5	1559.42	1557.97	1172.85	1259.84
6	1538.25	1542.76	1164.46	1256.84
7	1521.86	1532.05	1158.78	1250.13
8	1506.94	1523.55	1152.37	1247.82
9	1488.67	1515.96	1131.3	1239.69
10	1478.27	1509.63	1123.23	1235.43
11	1469.2	1505.82	1116.21	1229.84
12	1465.43	–^a^	1113.52	–^a^
13	1456.16	1499.01	1105.66	1221.84
14	1451.75	–^a^	1097.77	–^a^

^a^Due to convergence problems some structures were not analyzed

**Fig. 6:**
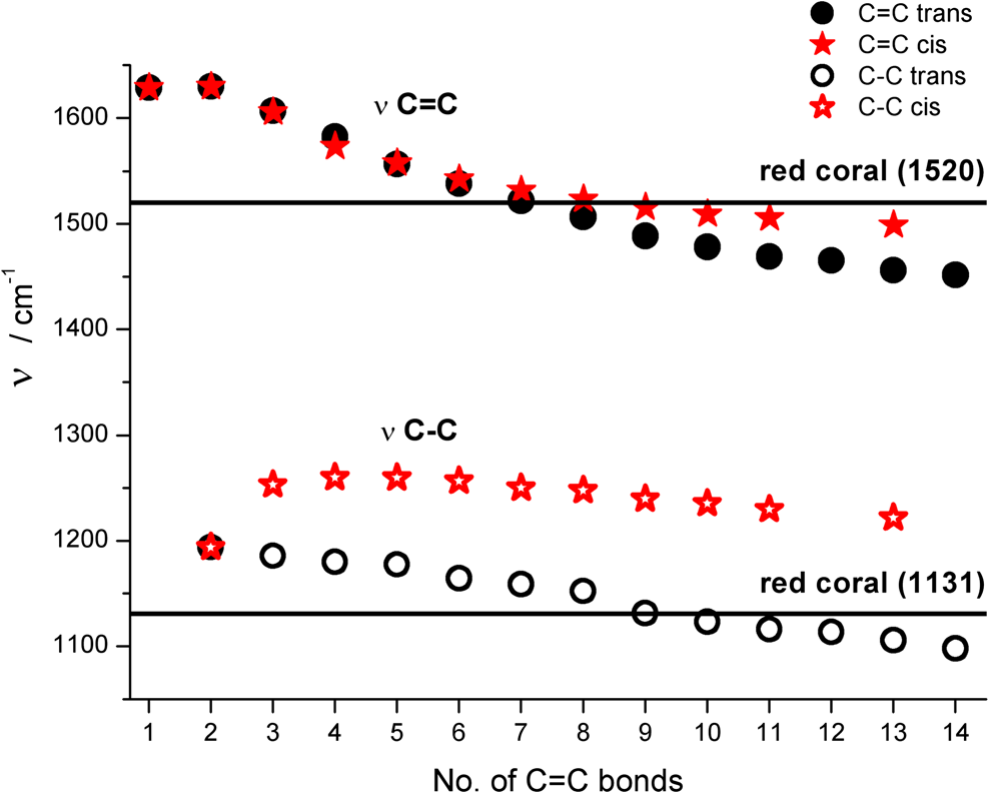
Sensitivity of Raman active single, and double carbon–carbon stretching frequencies (unscaled BLYP/6-311++G** results) to the length of all-trans and all-cis polyene chains. The observed band position in Raman spectra of red coral (continuous line) is also shown

It is interesting to notice nearly the same ν(C = C) values for both all-trans and all-cis polyenes for C1 to C7 members (see Table 2 and overlapped points in Fig. 6) and start differentiating for longer chains. For C13 polyenes, the observed difference between them is about 45 cm^−1^. A completely different picture is seen in the case of ν(C–C) values. Identical wavelengths are calculated only for C2 trans and cis isomers and, starting from C3, a widening of gap between the two isomers is observed. In case of all-cis C13 isomers its ν(C–C) values are significantly higher (by about 115 cm^−1^) than the vibration predicted for trans-isomer.

Using as a single diagnostic tool the predicted ν(C = C) value, the coral pigment should contain about 6–8 C = C units in their all-trans polyene pigments (see Fig. 6). However, taking into account the ν(C–C) vibrations only, the number of possible compounds could increase (from about C6 to C12). It is difficult to verify experimentally the performance of our simplified calculations for predicting stretching modes of polyenes. Obviously, the use of uniform scaling [45, 49, 50] of frequencies in vibrational spectra significantly improves the theoretical reproduction of experimental IR and Raman band positions. Moreover, a better but more expensive performance is observed by scaling individual vibrational modes (see also our recent studies on predicting harmonic and anharmonic frequencies in several small and medium size molecules [51–55]). On the other hand, it is apparent from different patterns of frequency changes of two signals observed in the predicted Raman spectra upon enlarging Cn that a different scaling factor should be used for each polyene molecule (Fig. 6).

**Fig. 7:**
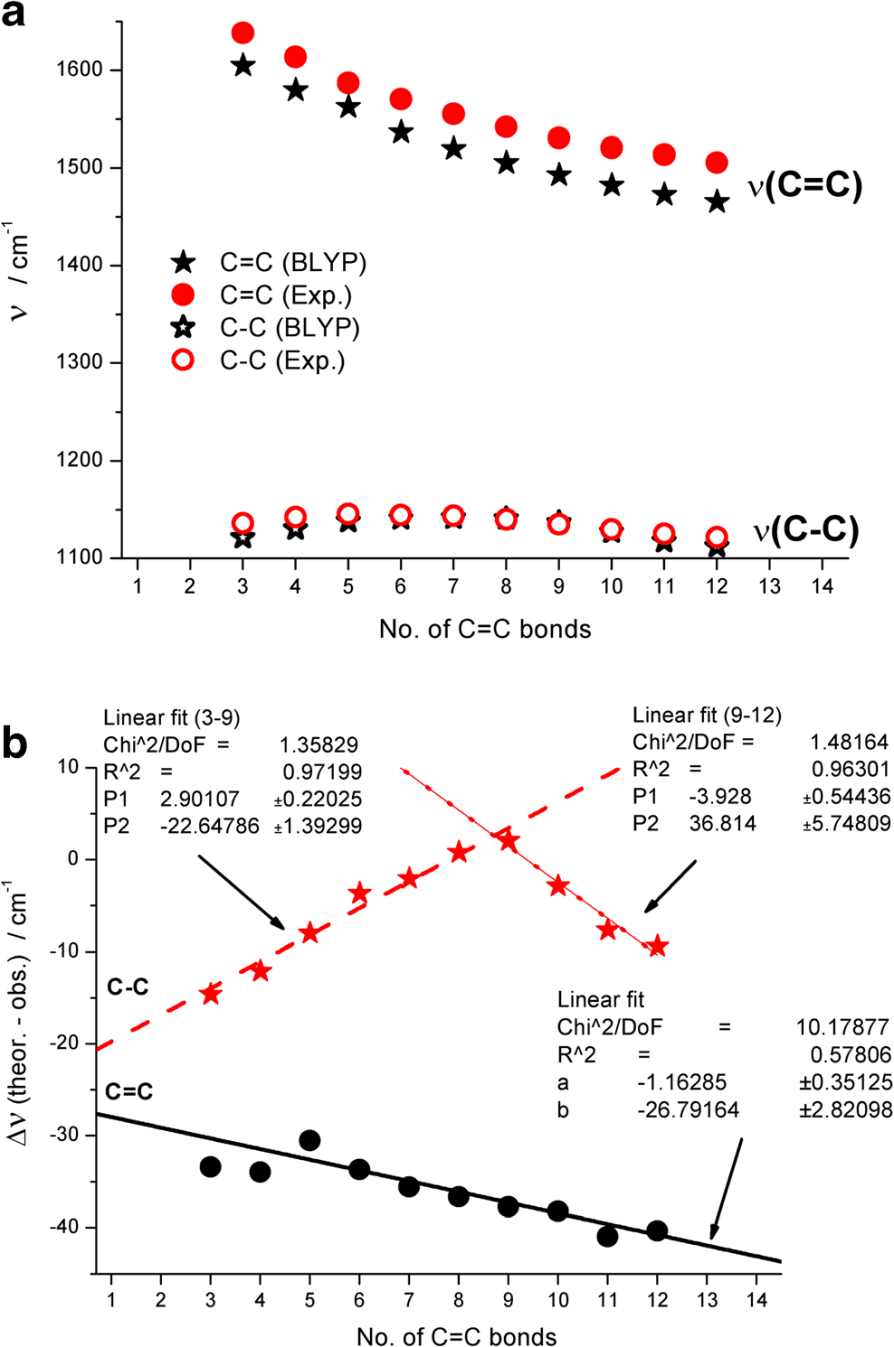
**a** Comparison of BLYP/6-311++G**-calculated (unscaled) harmonic ν(C = C) and ν(C–C) frequencies in all-trans tert-butyl ended polyenes containing 3 to 12 C = C bonds with experimental values. **b** Trends in deviations between B3LYP/6-311++G**-calculated harmonic ν(C = C) and ν(C–C) frequencies in all-trans tert-butyl ended polyenes vs. chain length. Linear fits of data points and fitting parameters are also shown

To make our analysis more accurate we selected an empirical approach by comparing our results with earlier reported experimental ν(C = C) and ν(C–C) values [3] for a set of C3–C12 all-trans polyenes capped at both ends with tert-butyl groups (Fig. 7a). The results plotted in Fig. 7a directly point out a very accurate prediction of ν(C–C) and significantly worse reproduction of ν(C = C) frequencies. In addition, both stretching modes are underestimated by BLYP/6-311++G** calculations (see Table S3). To explain this, one has to remember that high frequency vibrations, including O–H, N–H, C–H, and, to some degree, C = O are overestimated by theory [45, 49] (depending on the theory level it is sometimes 5 %, or even 10 %) but lower frequencies (below 1500 cm^−1^) can be slightly underestimated by theory.

The relatively small deviation of the calculated ν(C = C) and ν(C–C) stretching modes for C3–C12 all-trans polyenes capped with t-butyl groups from the experimental values [3] illustrates the accuracy of the applied BLYP/6-311++G** theory with respect to experiment, and shows some regular trends related to the length of the polyene chain (Fig. 7b). To be specific, a roughly linear change (y = −1.16285 *n −26.79164) of deviations from about −30 to about −40 cm^−1^ for ν(C = C) upon increasing the chain length is observed. However, in the case of ν(C–C) stretching mode, this deviation initially decreases from about −15 to 0 cm^−1^ (for C3–C9) and then, for C9–C12, drops to about −10 cm^−1^. In this case all data points for ν(C–C) were fitted with individual lines for two ranges of dimensions (y = 2.90107*n −22.64786 and y = −3.928*n + 36.814, respectively). The obtained linear fits will be used in a subsequent step as individually tuned correction factors for theoretical vibrations in both shorter and longer chains of all-trans polyenes.

**Fig. 8:**
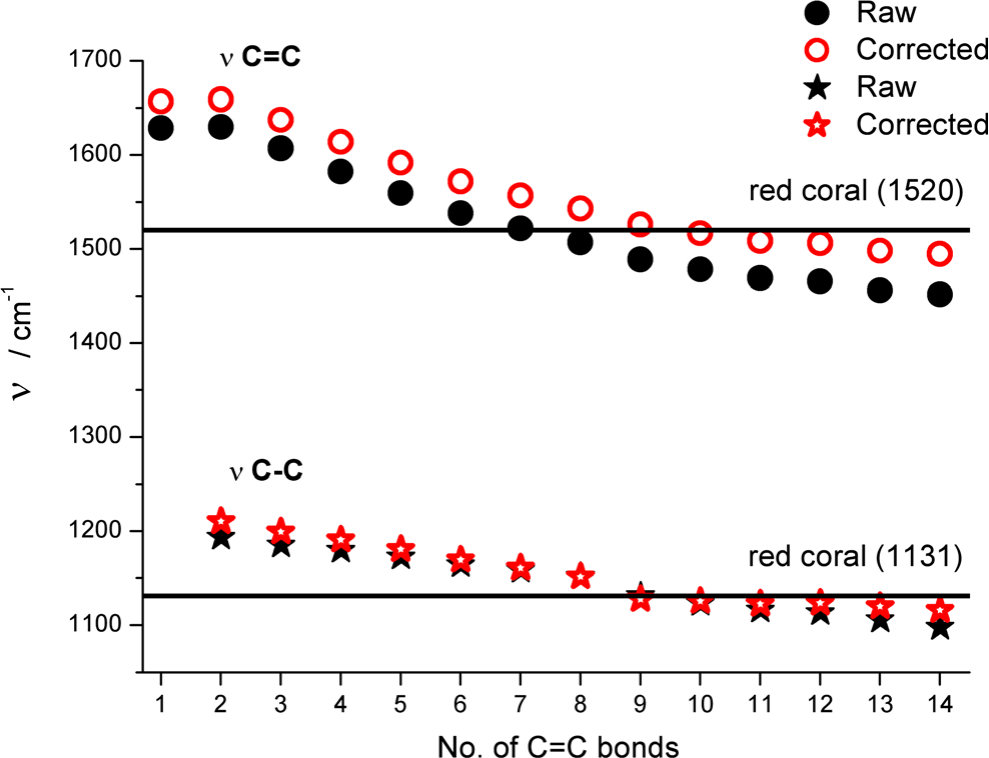
Comparison between raw and corrected B3LYP/6-311++G** calculated harmonic ν(C = C) and ν(C–C) frequencies in ethylene and all-trans C2 – C14 polyenes with reported Raman bands position in red coral pigment

Figure 8 plots BLYP/6-311++G**-calculated ν(C = C) and ν(C–C) data of the studied unsubstituted C1–C14 systems (Fig. 6) improved with linear correcting factors derived from Fig. 7 (see also Table S4). It is apparent from Fig. 8 that the corrected points for ν(C = C) and ν(C–C) stretching modes are less sensitive to Cn and saturate faster (at about C8–C9) in comparison to raw calculated data. Starting from this chain length of all-trans polyenes, both frequencies are fairly close to positions of experimental bands observed for coral. Assuming a safe error margin of ±20 and ±10 cm^−1^ for ν(C = C) and ν(C–C), one could conclude from Fig. 8 that red coral pigment contains 9–12 C = C units.

## Conclusions

Systematic BLYP and B3LYP calculations combined with 6-311++G** basis set of structural parameters and BLYP/6-311++G** harmonic frequencies of ethylene and all-trans and all-cis polyenes containing up to 14 conjugated C = C bonds were performed. For the first time, the convergence of structural and vibrational parameters was observed upon enlarging the chain length starting from ethylene to 14 C = C units. The suitability of BLYP-calculated harmonic frequencies for assessment of all-trans polyene chain length was shown, as well as scaled vs. experimental data for tert-butyl capped polyenes containing 3–12 C = C units .

The presence of all-trans polyenes containing 9–12 C = C double bond units in red coral pigment was concluded from comparison of the theoretical frequencies of the two most intense bands due to ν(C = C) and ν(C–C) stretching vibrations with reported experimental band positions.

In analogy to linear acenes, future theoretical studies on all-trans and all-cis polyenes of controlled size should also concentrate on modeling their stability, HOMO-LUMO energy and optical properties, as well as observation of convergence patterns of their characteristic parameters. A knowledge of these properties would be important for prediction of their photochemical parameters and their potential applications in material science and electronics.

## Electronic supplementary material

Below is the link to the electronic supplementary material.

ESM 1 (DOC 2851 kb)
