# Supplementary material for: DFT studies on the structural and vibrational properties of polyenes
Source: J Mol Model. 2016 Apr 6;22:101. doi: 10.1007/s00894-016-2969-1 (PMC4821862; doi:10.1007/s00894-016-2969-1)
Supplement: Supplementary file 1 — (DOC 2851 kb) [file 894_2016_2969_MOESM1_ESM.doc]

**Supplementary material for:**

**DFT studies on structural and vibrational properties of polyenes**

Teobald Kupka*, Aneta Buczek*, Małgorzata A. Broda, Michał Stachów, Przemysław Tarnowski

^a^Faculty of Chemistry, University of Opole, 48, Oleska Street, 45-052 Opole, Poland


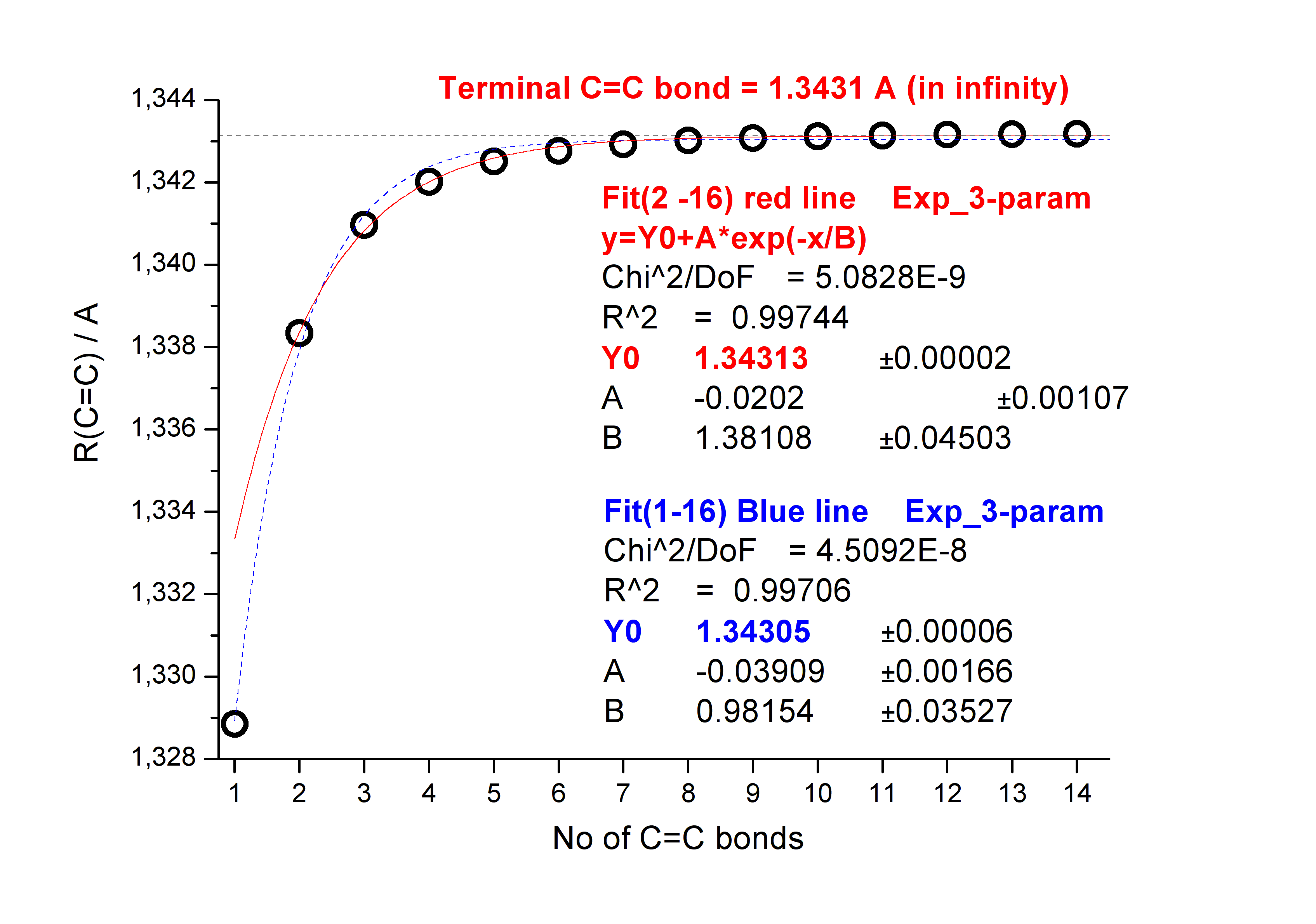
**Figure S1A**. Convergence of B3LYP/6-311++G** calculated terminal C=C bond length in all-trans polyenes with 1 to 14 conjugated double bond units. Results of three-parameter fits using 1-14 (blue dashed line) or 2-14 data points (red continuous line) are shown


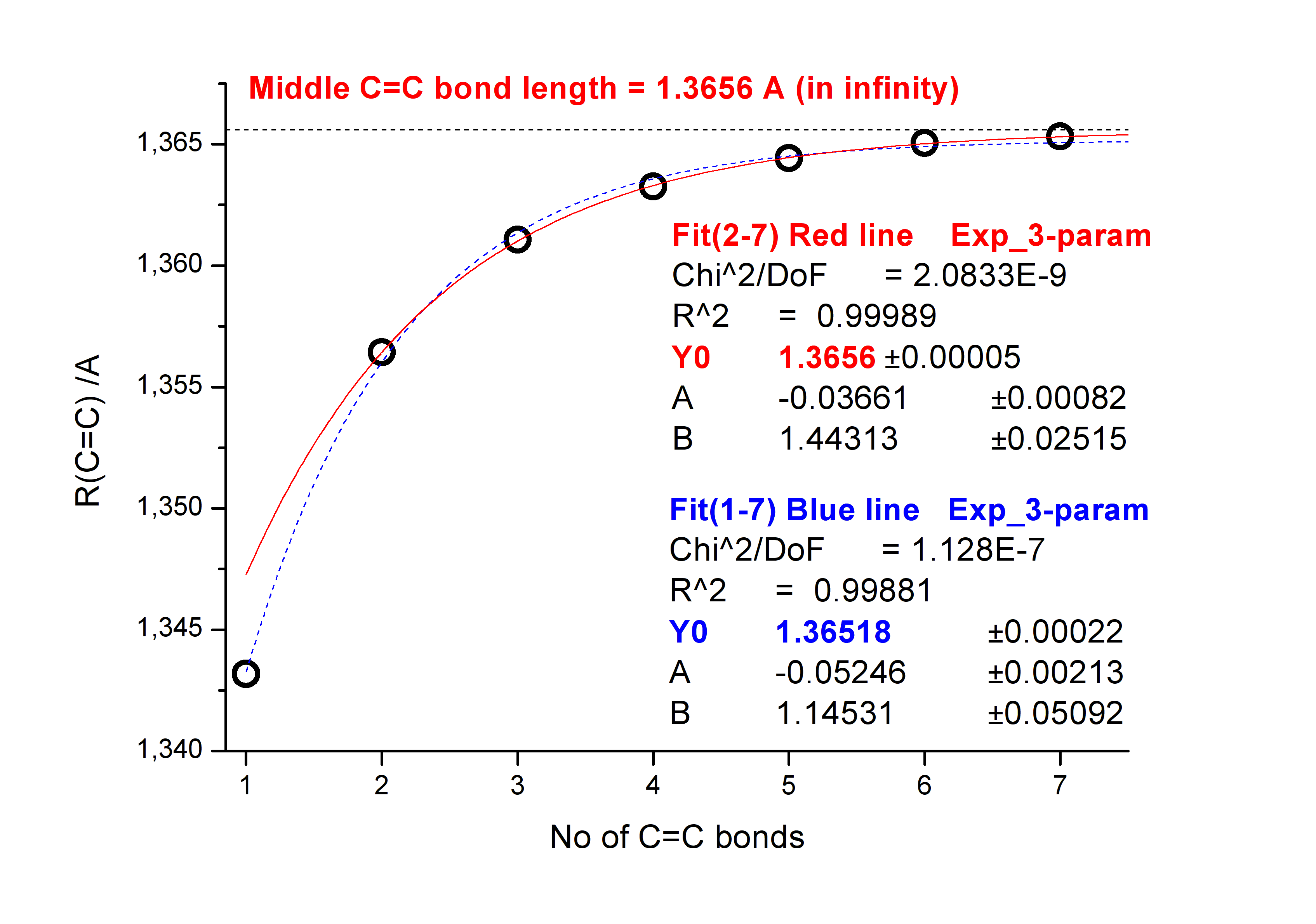
**Figure S1B.** Convergence of B3LYP/6-311++G** calculated C=C bond length in the middle of all-trans polyene chains with 1 to 14 conjugated double bond units. Results of three-parameter fits using 1-7 (blue dashed line) or 2-7 data points (red continuous line) are shown


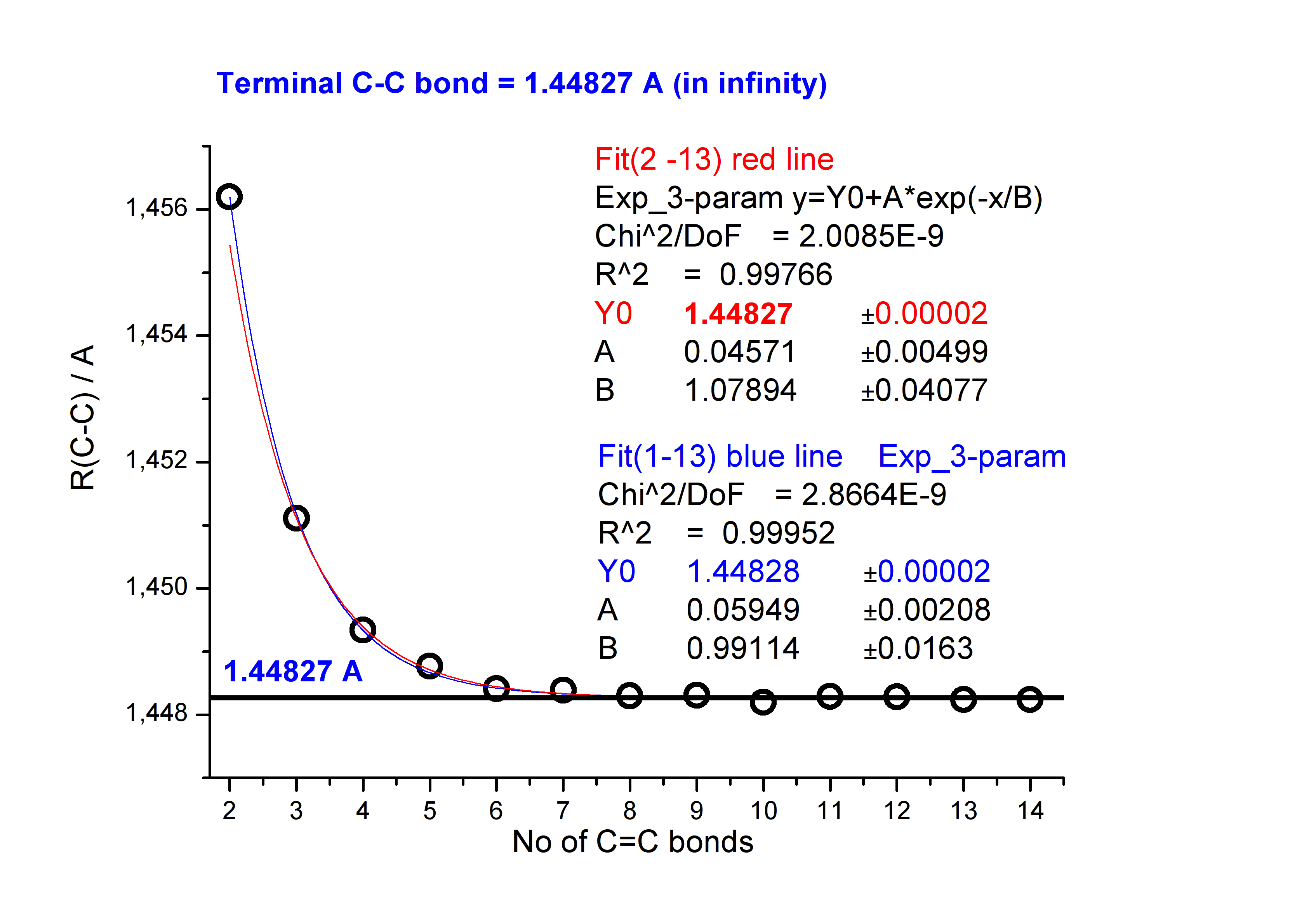
**Figure S1C**. Convergence of B3LYP/6-311++G** calculated terminal C-C bond length in all-trans polyenes with 1 to 14 conjugated double bond units. Results of three-parameter fits using 1-14 (blue dashed line) or 2-14 data points (red continuous line) are shown


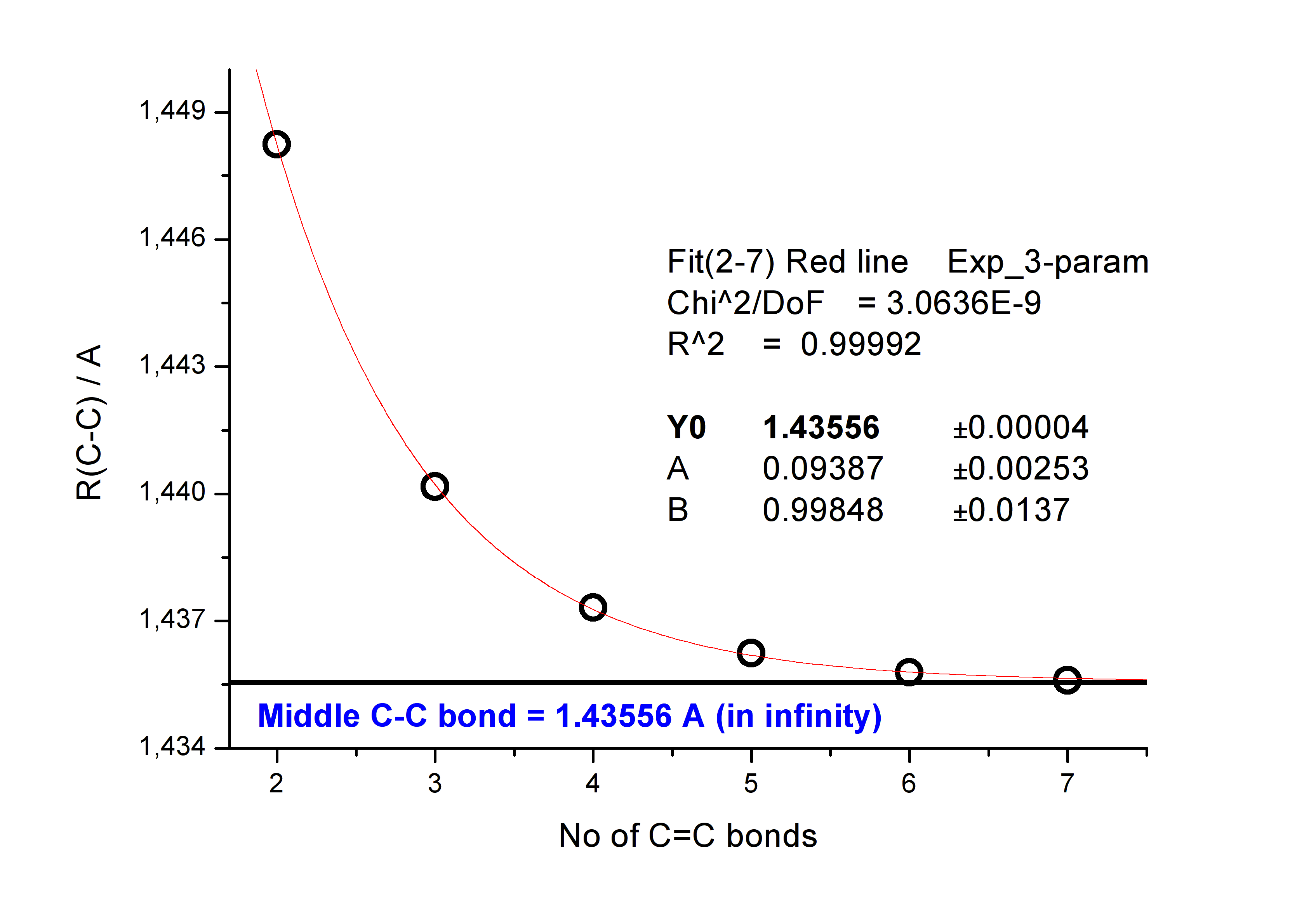
**Figure S1D.** Convergence of B3LYP/6-311++G** calculated C-C bond length in the middle of all-trans polyene chains with 1 to 14 conjugated double bond units. Results of three-parameter fits using 2-6 data points (red continuous line) are shown


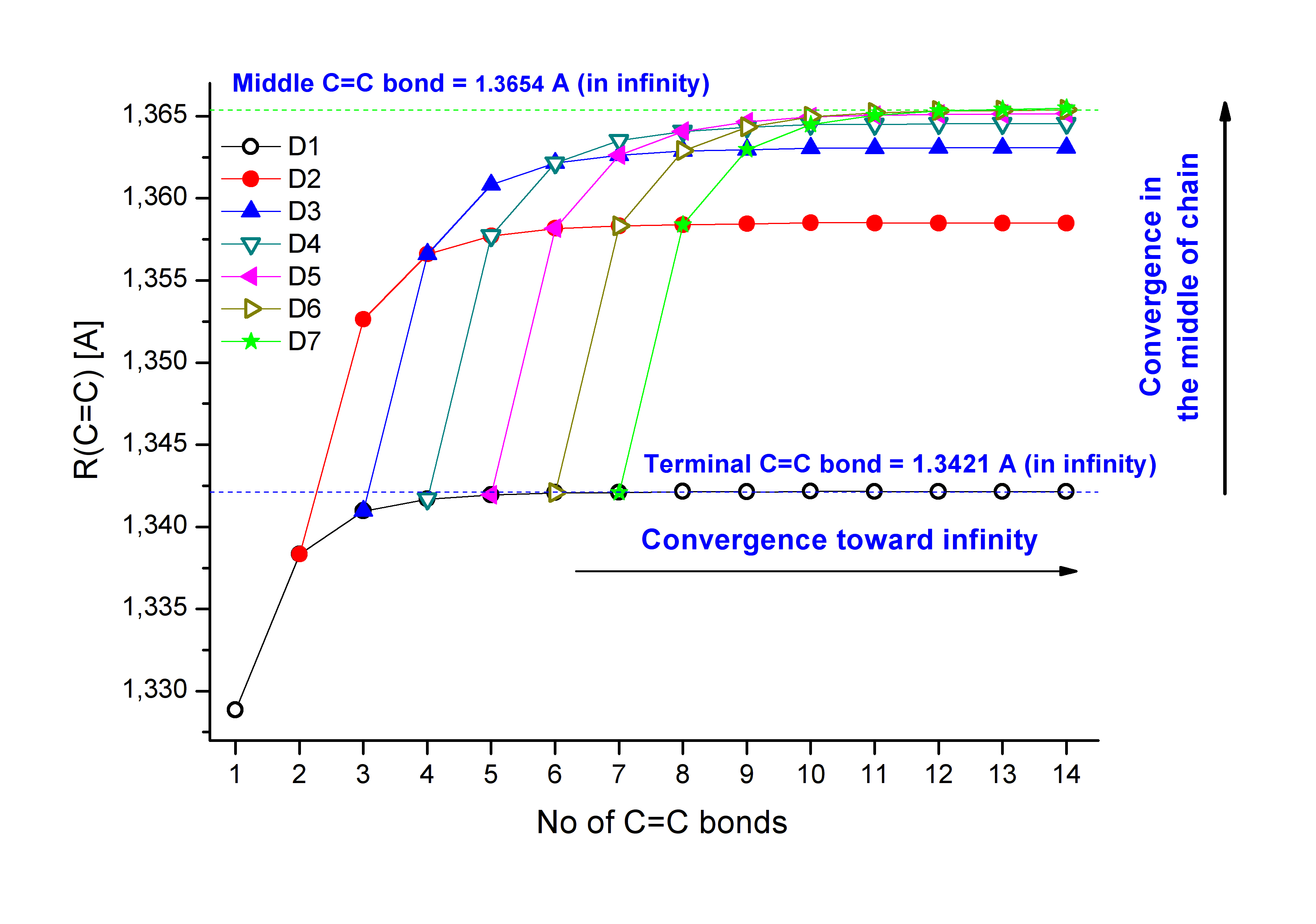
**Figure S2A.** Changes of B3LYP/6-311++G** calculated C=C bond length in all-cis polyenes with 1 to 14 conjugated double bond units. Convergence patterns of bond length with increasing chain dimension are indicated


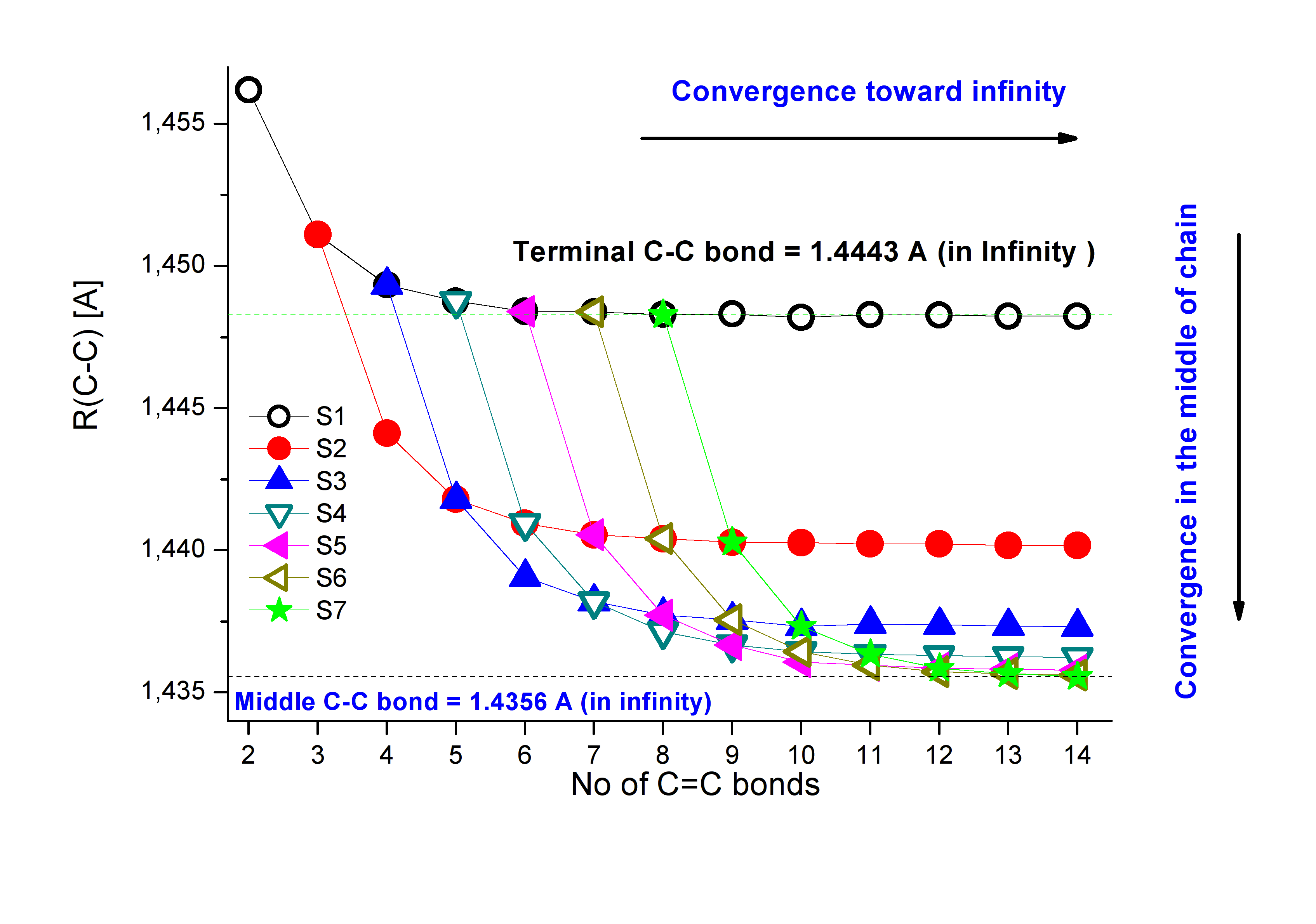
**Figure S2B.** Changes of B3LYP/6-311++G** calculated C-C bond length in all-cis polyenes with 1 to 14 conjugated double bond units. Convergence patterns of bond length with increasing chain dimension are indicated


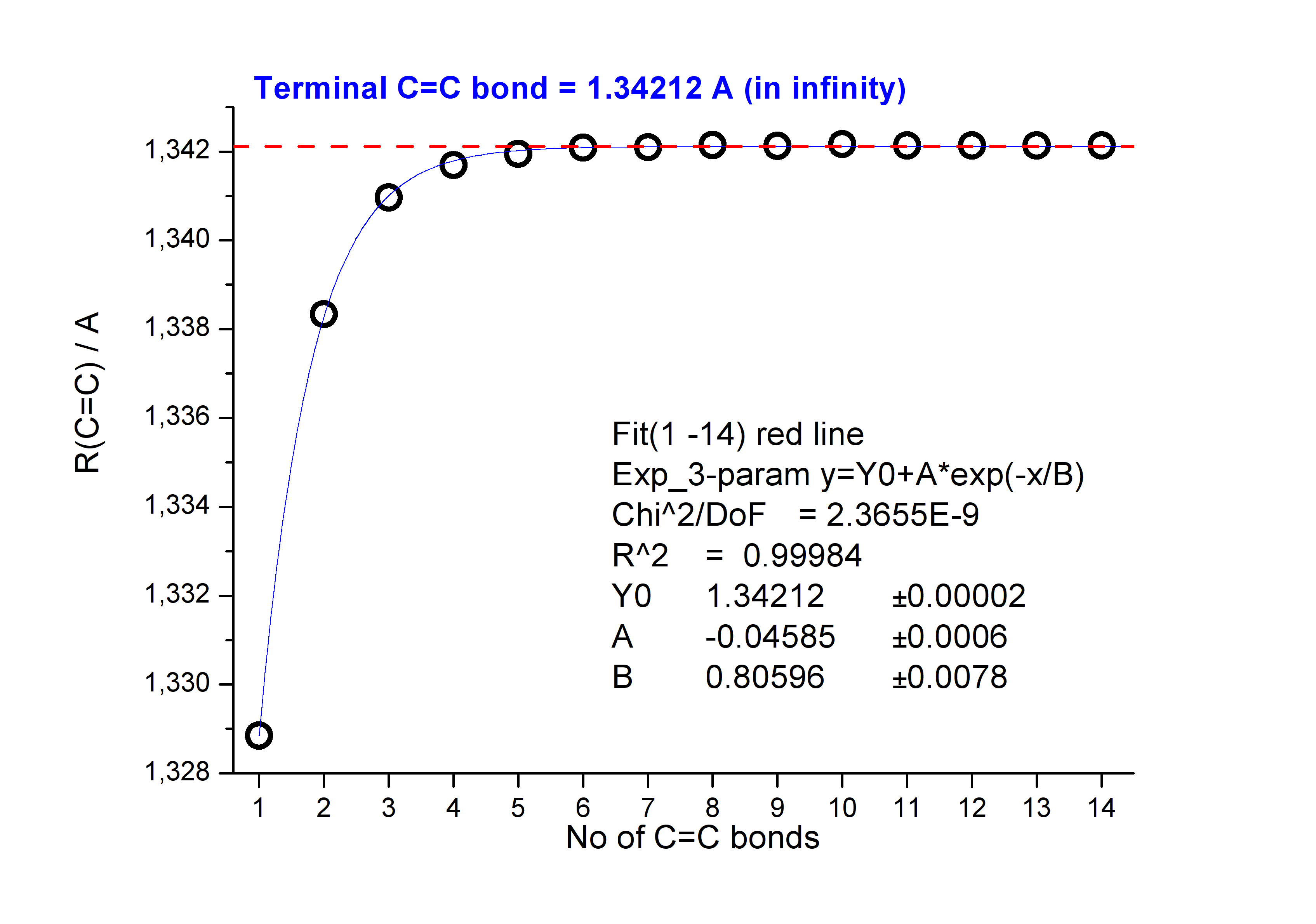
**Figure S3A.** Convergence of B3LYP/6-311++G** calculated terminal C=C bond length in all-cis polyenes with 1 to 14 conjugated double bond units. Results of three-parameter fits using 1-14 data points is shown (blue dashed line)


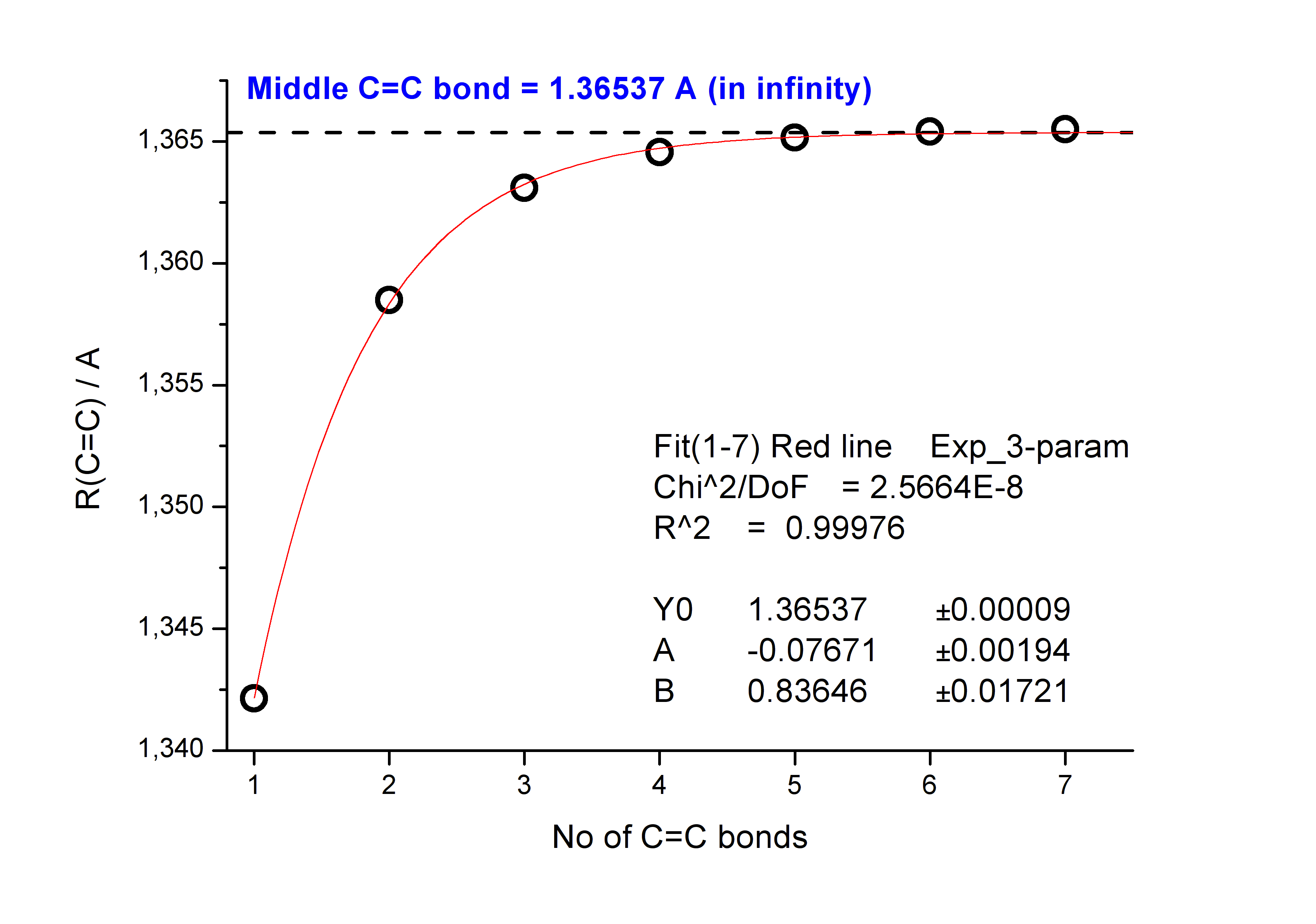
**Figure S3B.** Convergence of B3LYP/6-311++G** calculated C=C bond length in the middle of all-cis polyene chains with 1 to 14 conjugated double bond units. The result of three-parameter fits using 1-7 data points is shown (red continuous line)


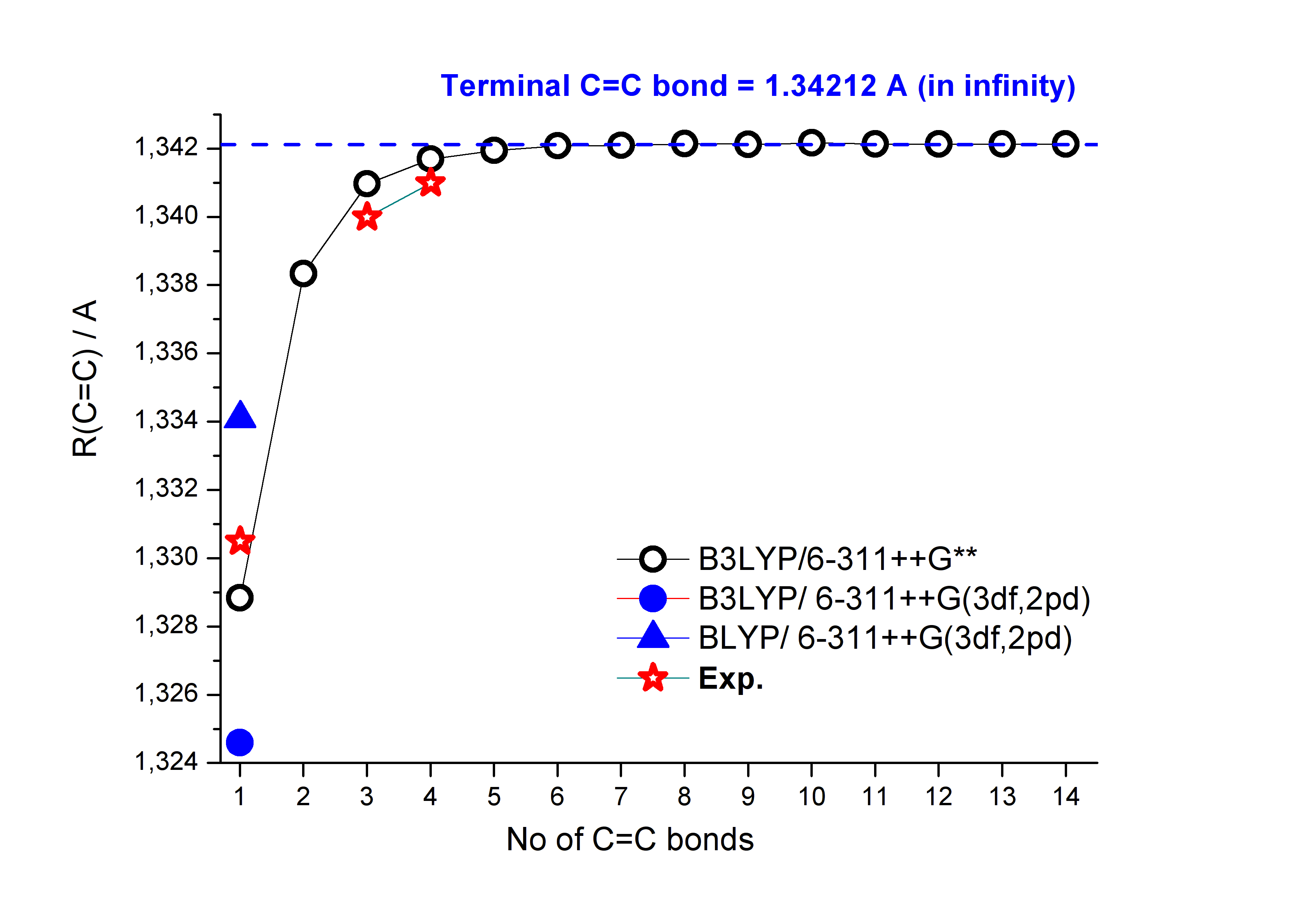
**Figure S3C.** Convergence of B3LYP/6-311++G** calculated terminal C=C bond length in all-cis polyenes with 1 to 14 conjugated double bond units. Available experimental and benchmark theoretical results are also shown.


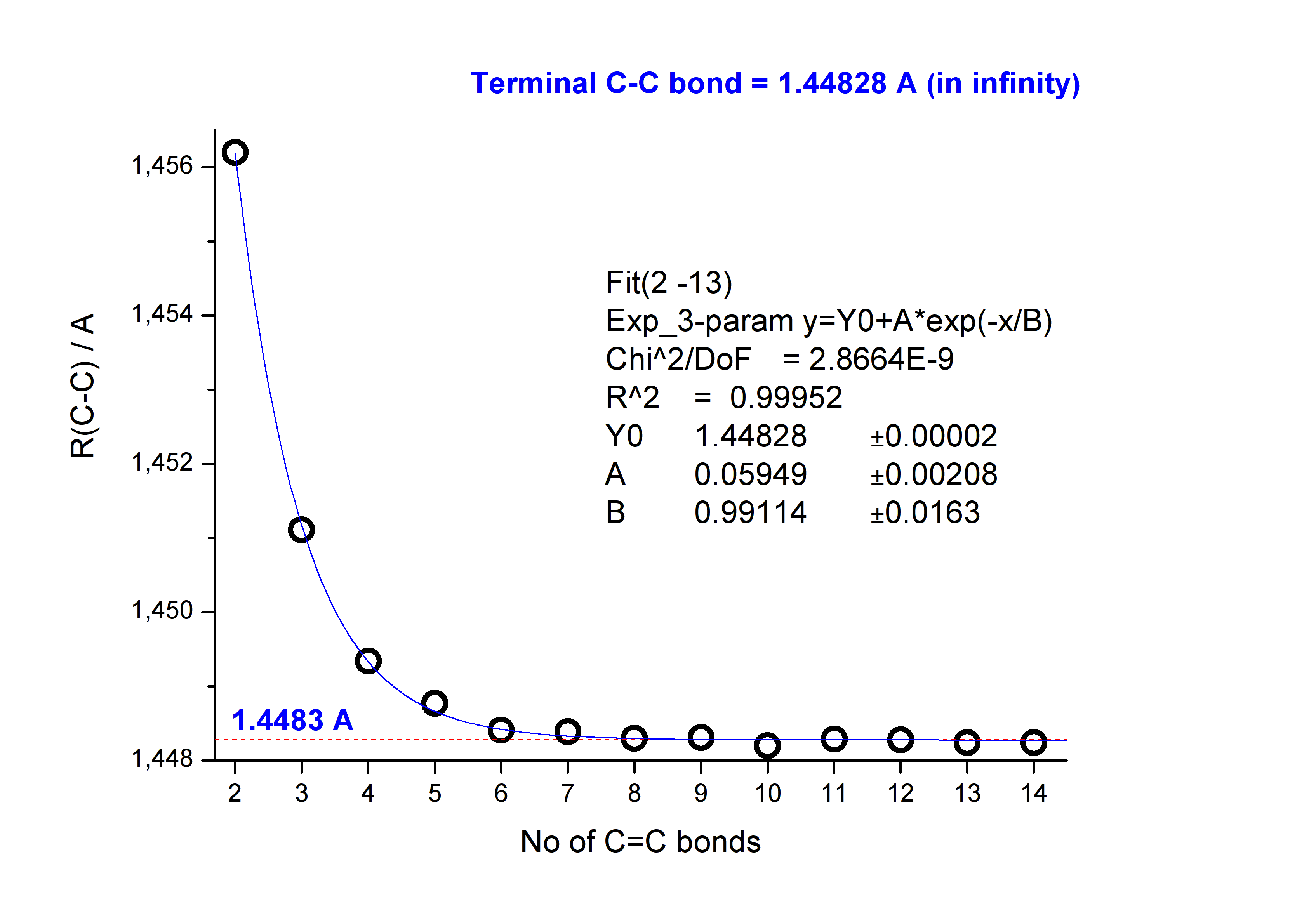
**Figure S3D.** Convergence of B3LYP/6-311++G** calculated terminal C-C bond length in all-cis polyenes with 1 to 14 conjugated double bond units. Results of three-parameter fits using 1-14 data points and the value for very long chain are shown (blue and red lines)


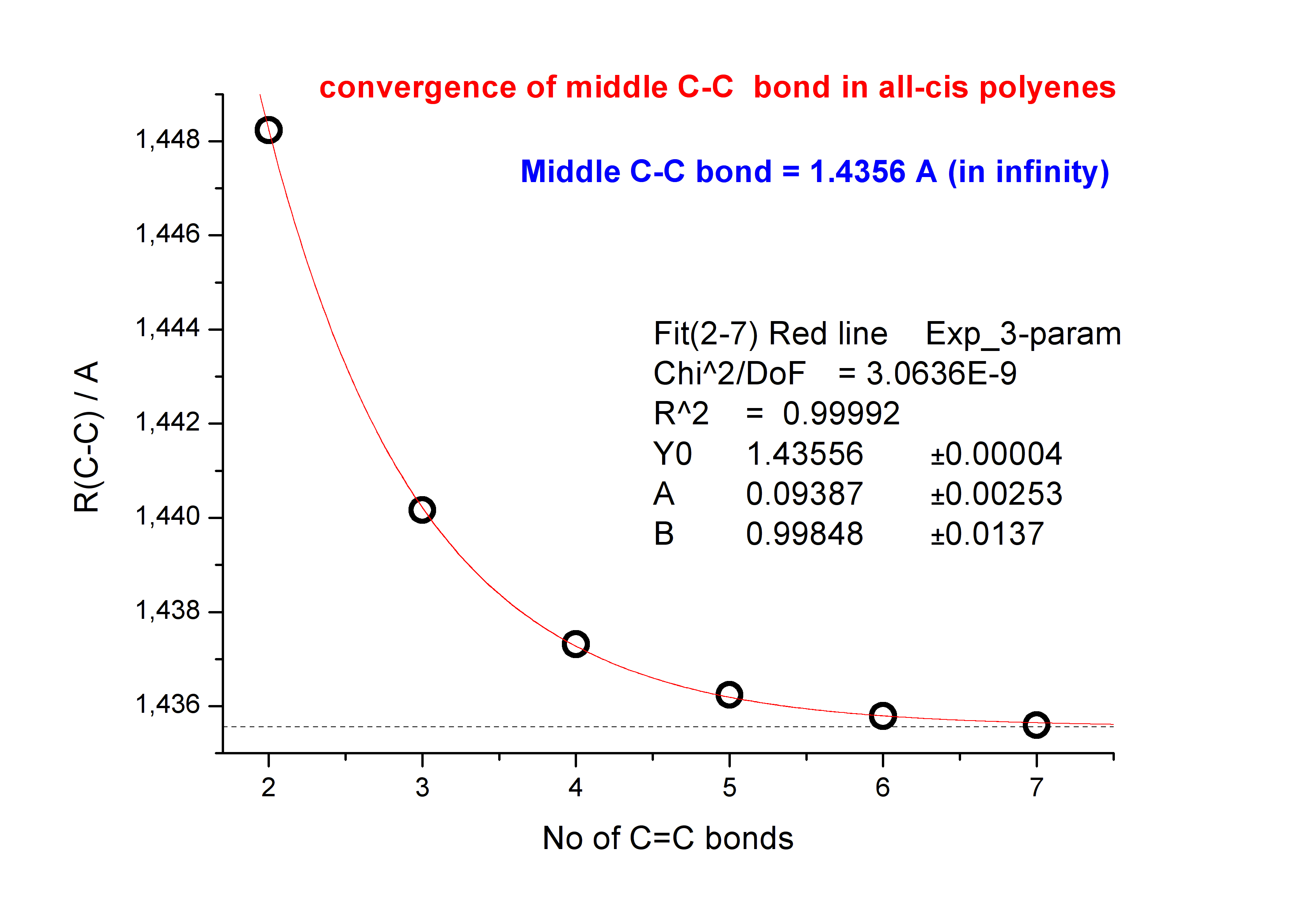
**Figure S3E.** Convergence of B3LYP/6-311++G** calculated C-C bond length in the middle of all-cis polyene chains with 1 to 14 conjugated double bond units. Results of three-parameter fits using 1-7 data points and the value for very long chain are shown (red and blue lines)


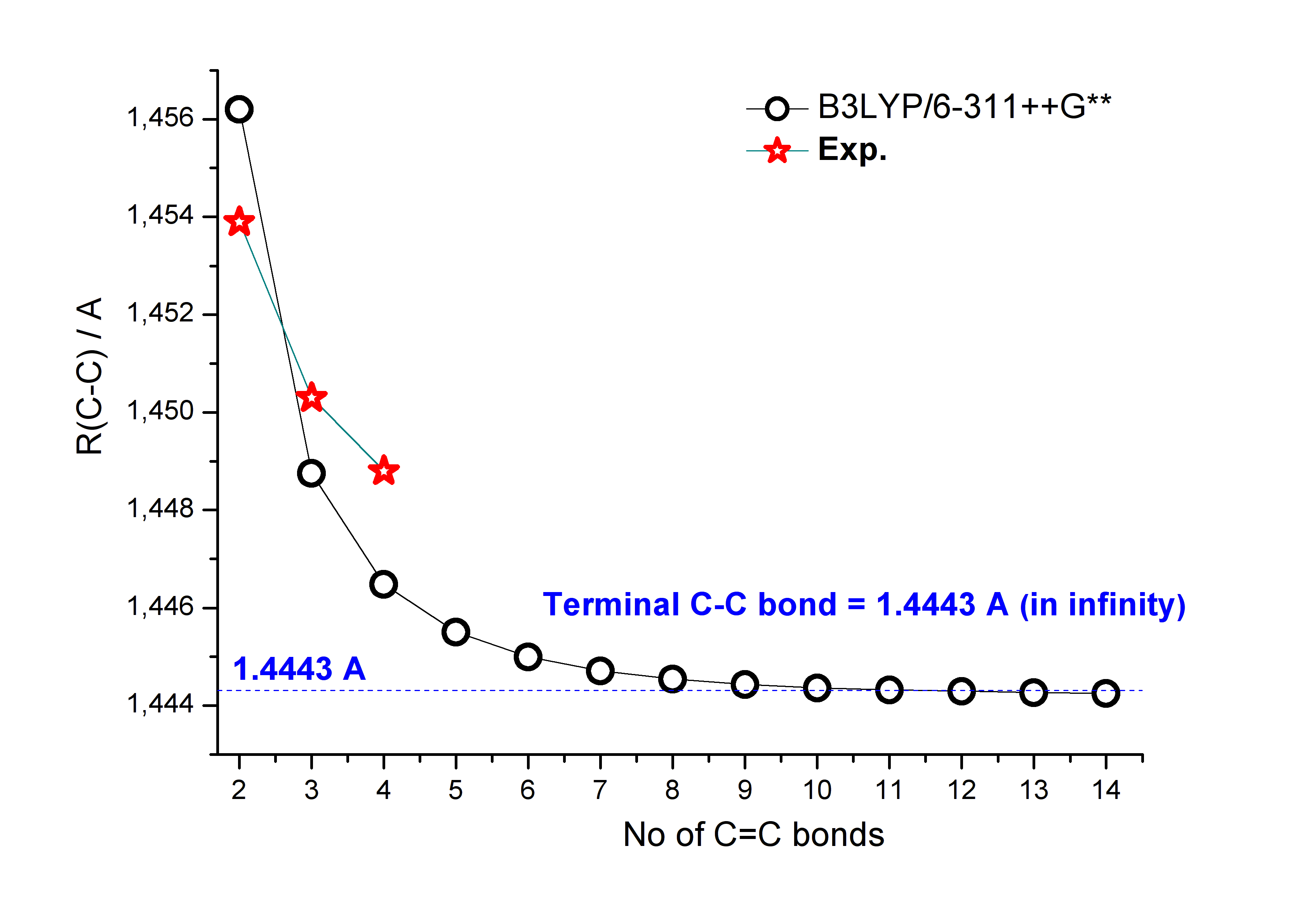
**Figure S3F.** Convergence of B3LYP/6-311++G** calculated terminal C-C bond length in all-cis polyenes with 1 to 14 conjugated double bond units. Available experimental and benchmark theoretical results are also shown


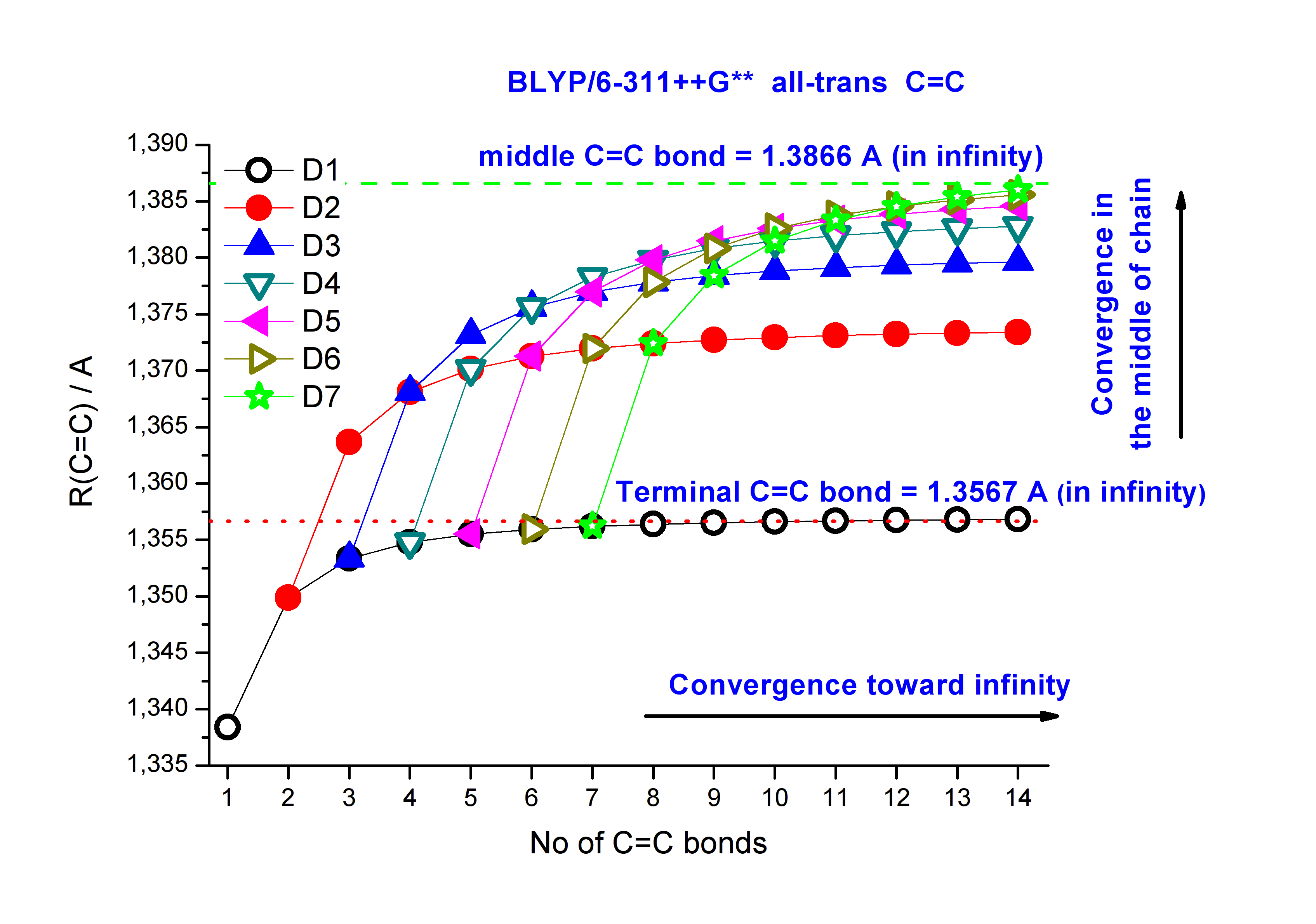
Figure S4A. Change of BLYP/6-311++G** calculated C=C bond length in all-trans polyenes with 1 to 14 conjugated double bond units. Convergences of terminal C=C bond length and in the middle of molecule with increasing chain length are indicated


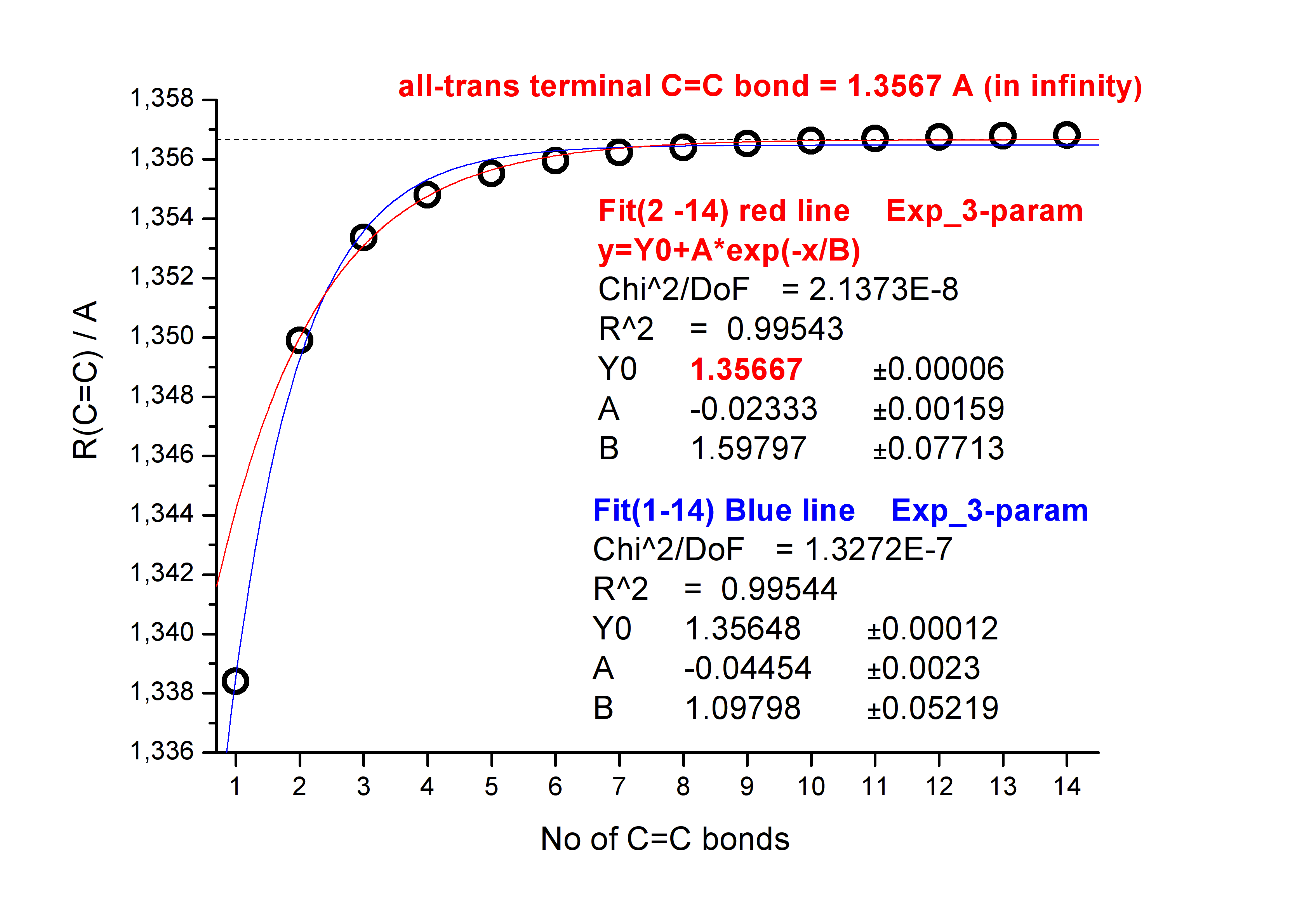
Figure S4B. Convergence of BLYP/6-311++G** calculated C=C bond length in the middle of all-cis polyene chains with 1 to 14 conjugated double bond units. The result of three-parameter fits using 1-14 and 2-14 data points is shown (blue and red continuous lines)


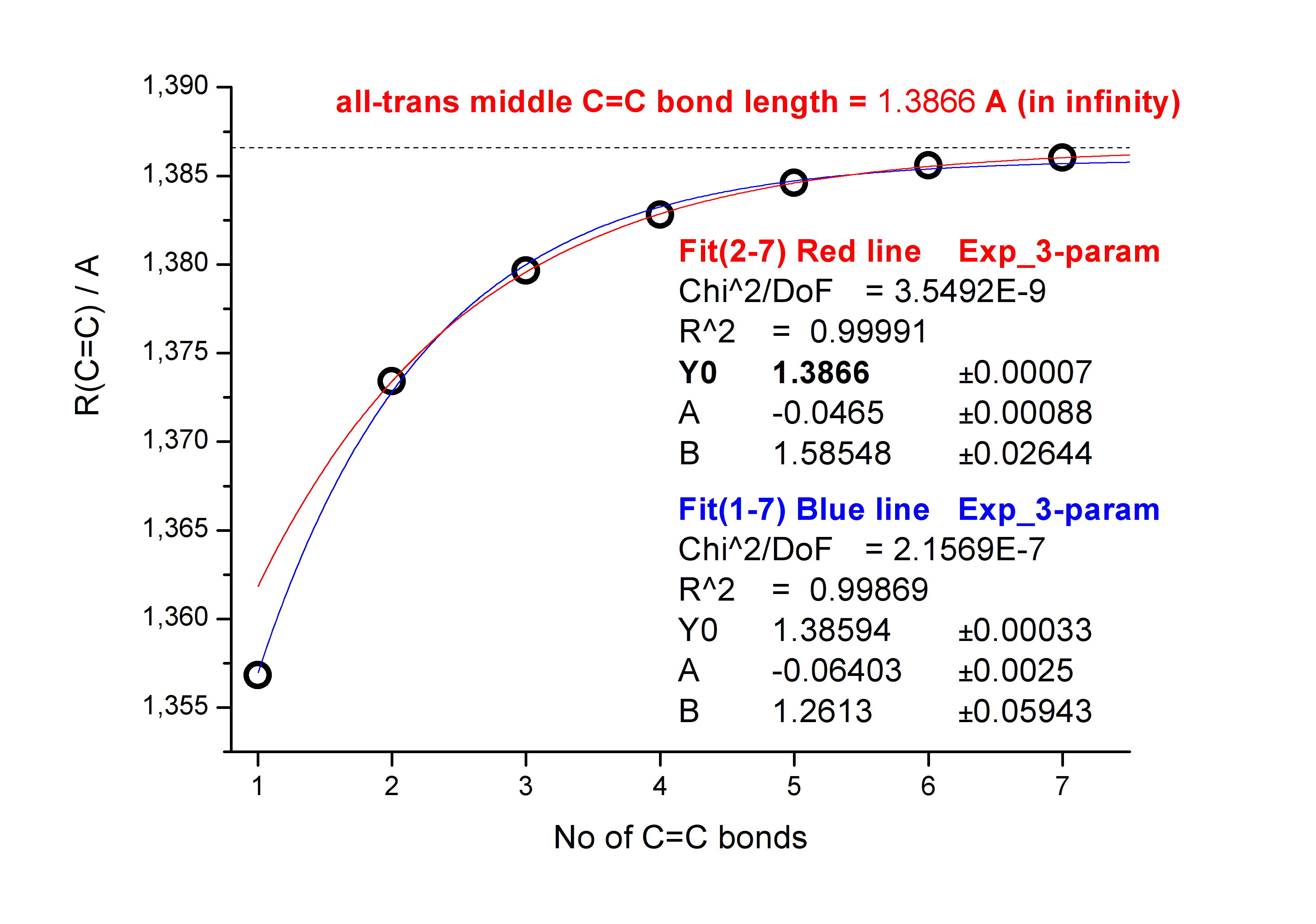
Figure S4C. Convergence of BLYP/6-311++G** calculated C=C bond length in the middle of all-trans polyene chains with 1 to 14 conjugated double bond units. The result of three-parameter fits using 1-7 and 2-7 data points is shown (blue and red continuous lines)


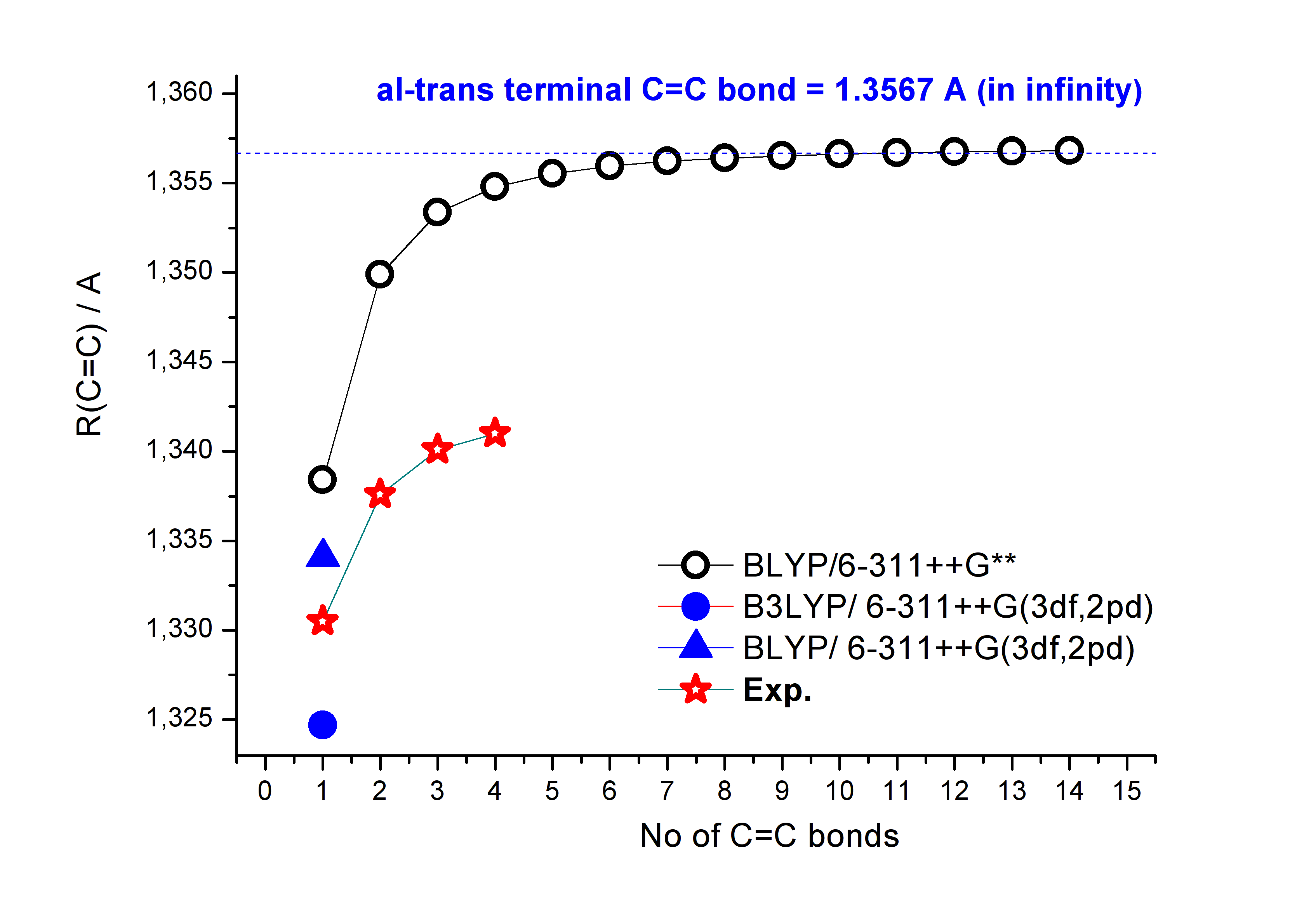
Figure S4D. Convergence of BLYP/6-311++G** calculated terminal C=C bond length in all-trans polyenes with 1 to 14 conjugated double bond units. Available experimental and benchmark theoretical results are also shown


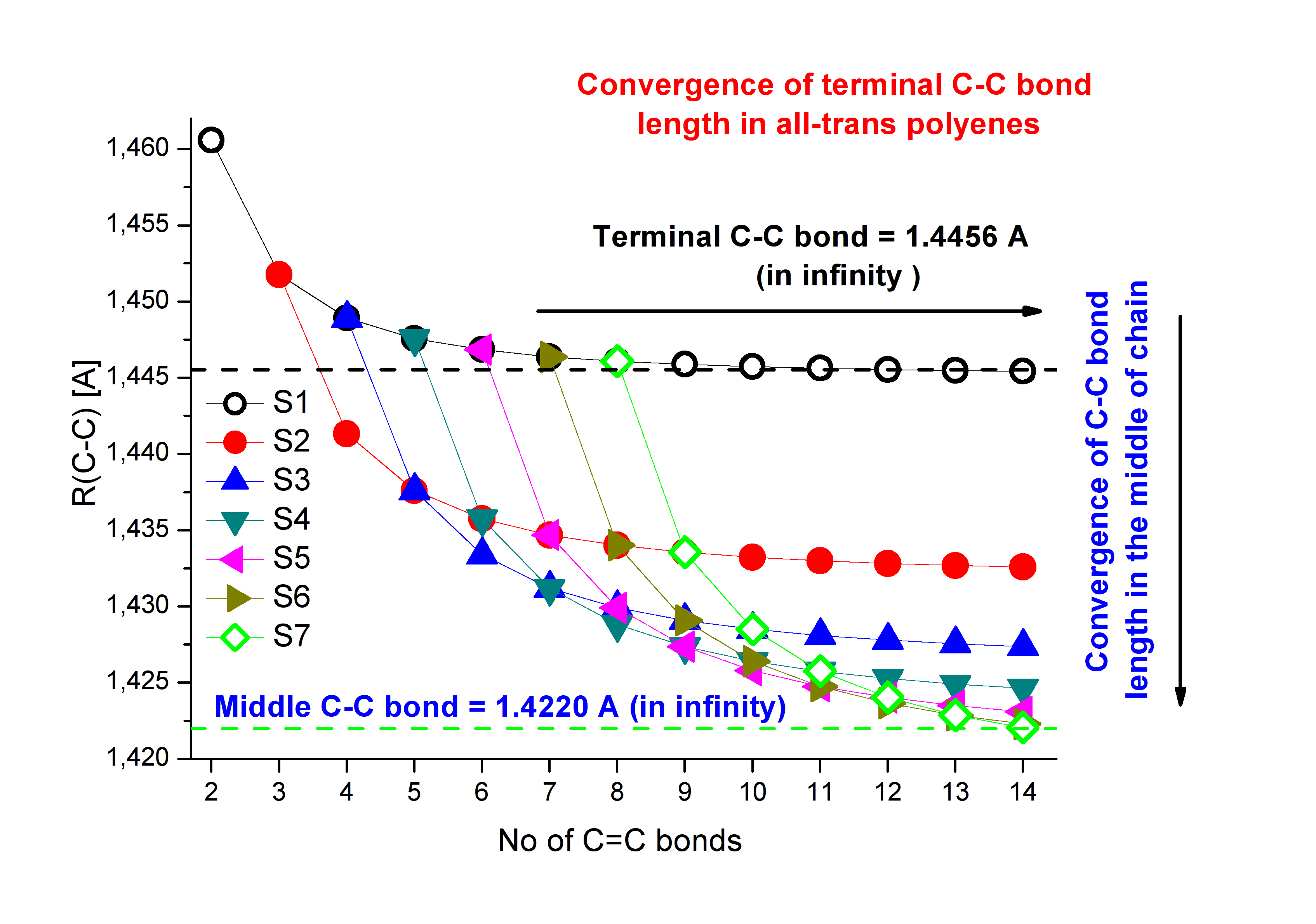
Figure S4E. Change of BLYP/6-311++G** calculated C-C bond length in all-trans polyenes with 1 to 14 conjugated double bond units. Convergences of terminal C=C bond length and in the middle of molecule with increasing chain length are indicated


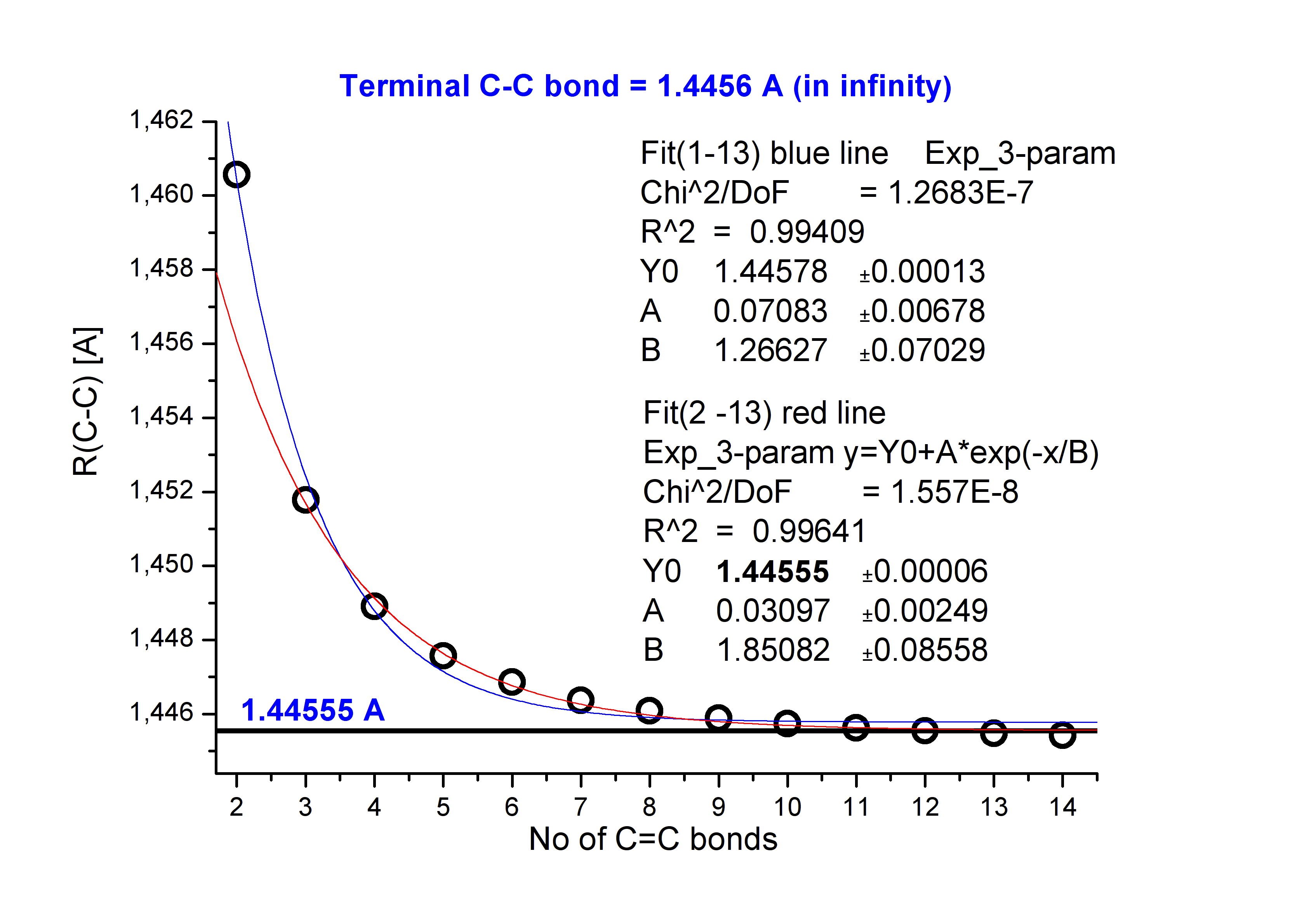
Figure S4F. Convergence of BLYP/6-311++G** calculated C-C bond length in the middle of all-cis polyene chains with 1 to 14 conjugated double bond units. The result of three-parameter fits using 1-14 and 2-14 data points are shown (blue and red continuous lines)


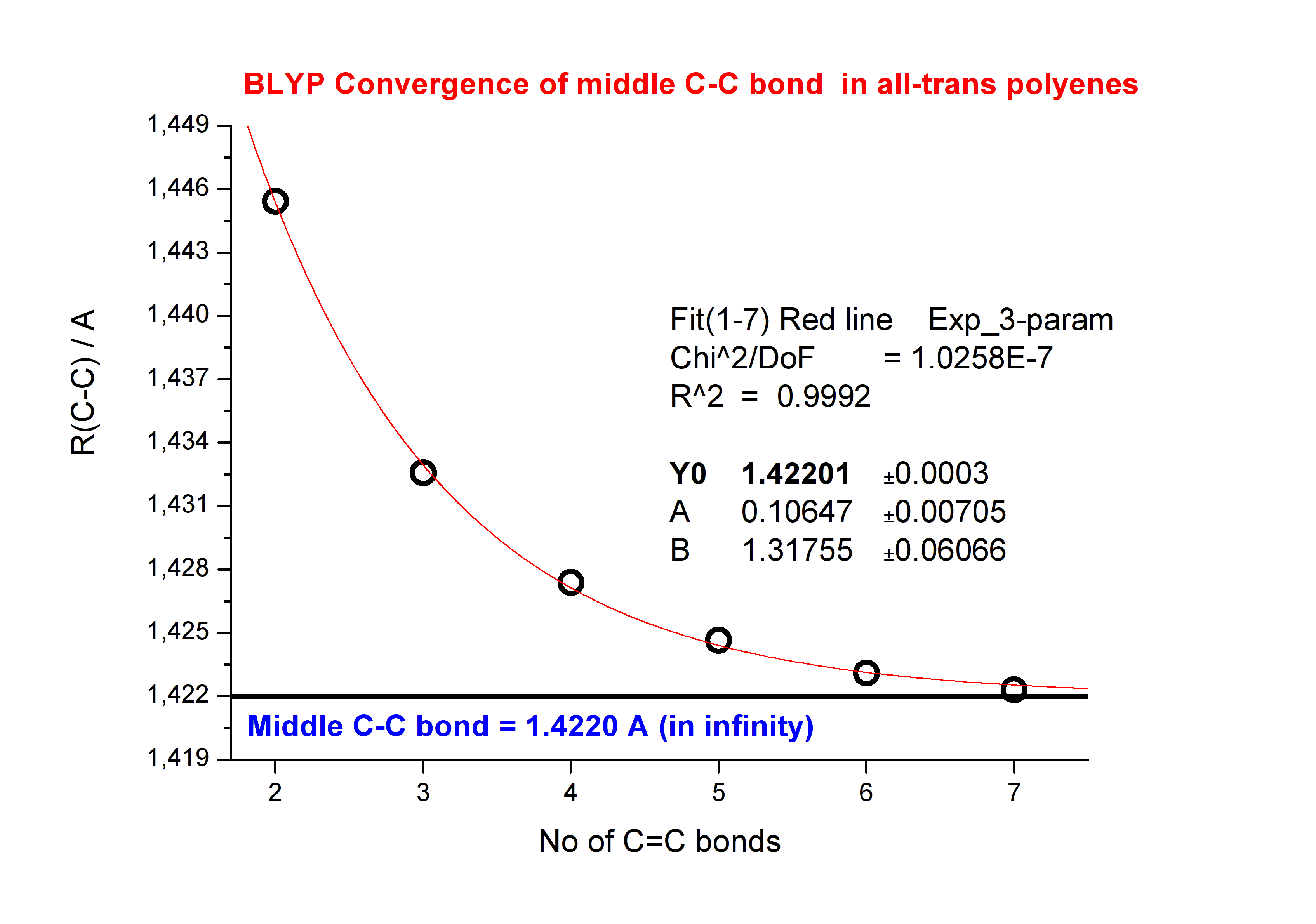
Figure S4G. Convergence of BLYP/6-311++G** calculated C-C bond length in the middle of all-trans polyene chains with 1 to 14 conjugated double bond units. The result of three-parameter fits using 1-6 data points is shown (red curve)


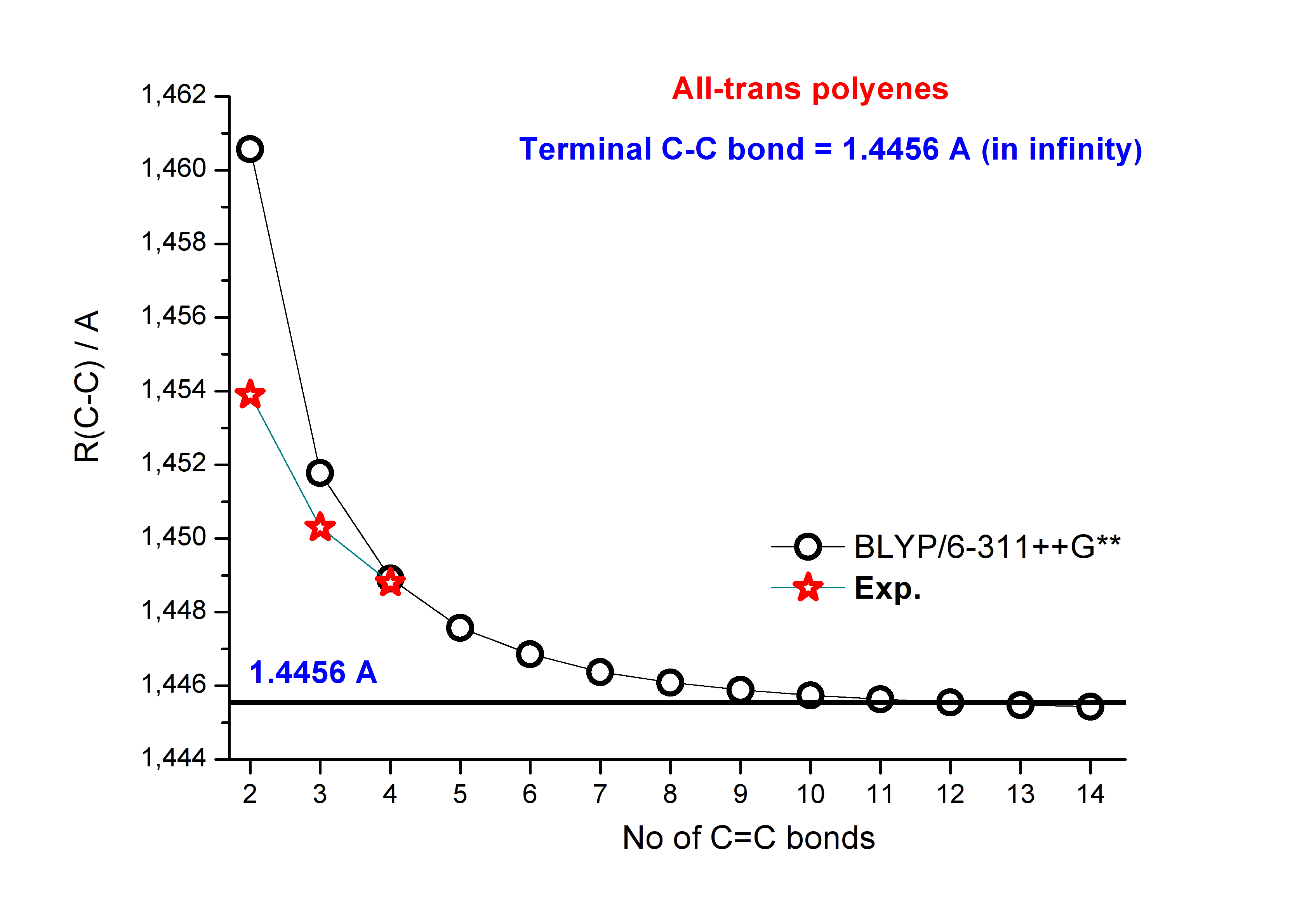
Figure S4H. Convergence of BLYP/6-311++G** calculated terminal C-C bond length in all-trans polyenes with 1 to 14 conjugated double bond units. Available experimental and benchmark theoretical results are also shown


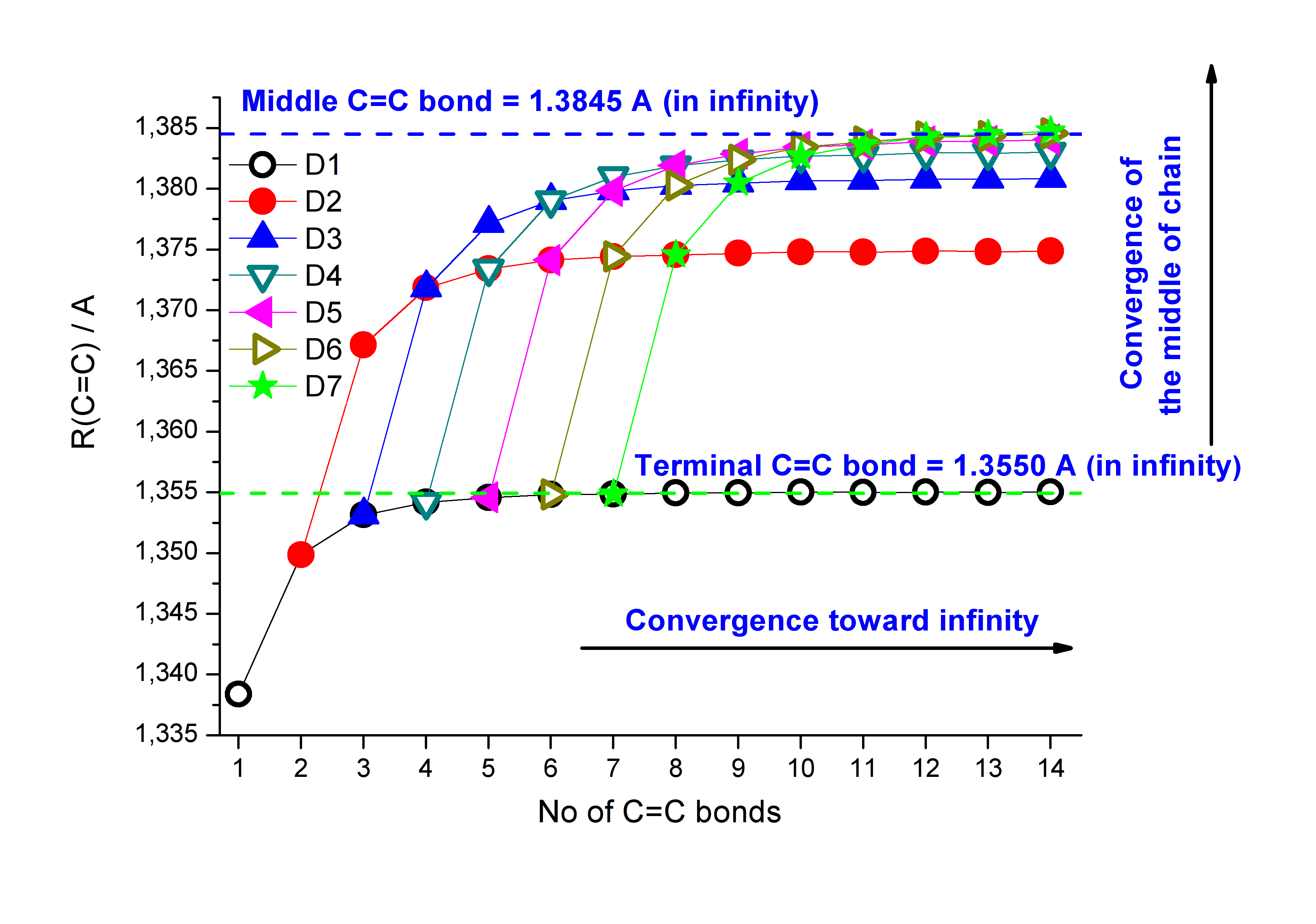
Figure S5A. Convergence of BLYP/6-311++G** calculated C=C bond length in all-cis polyene chains with 1 to 14 conjugated double bond units


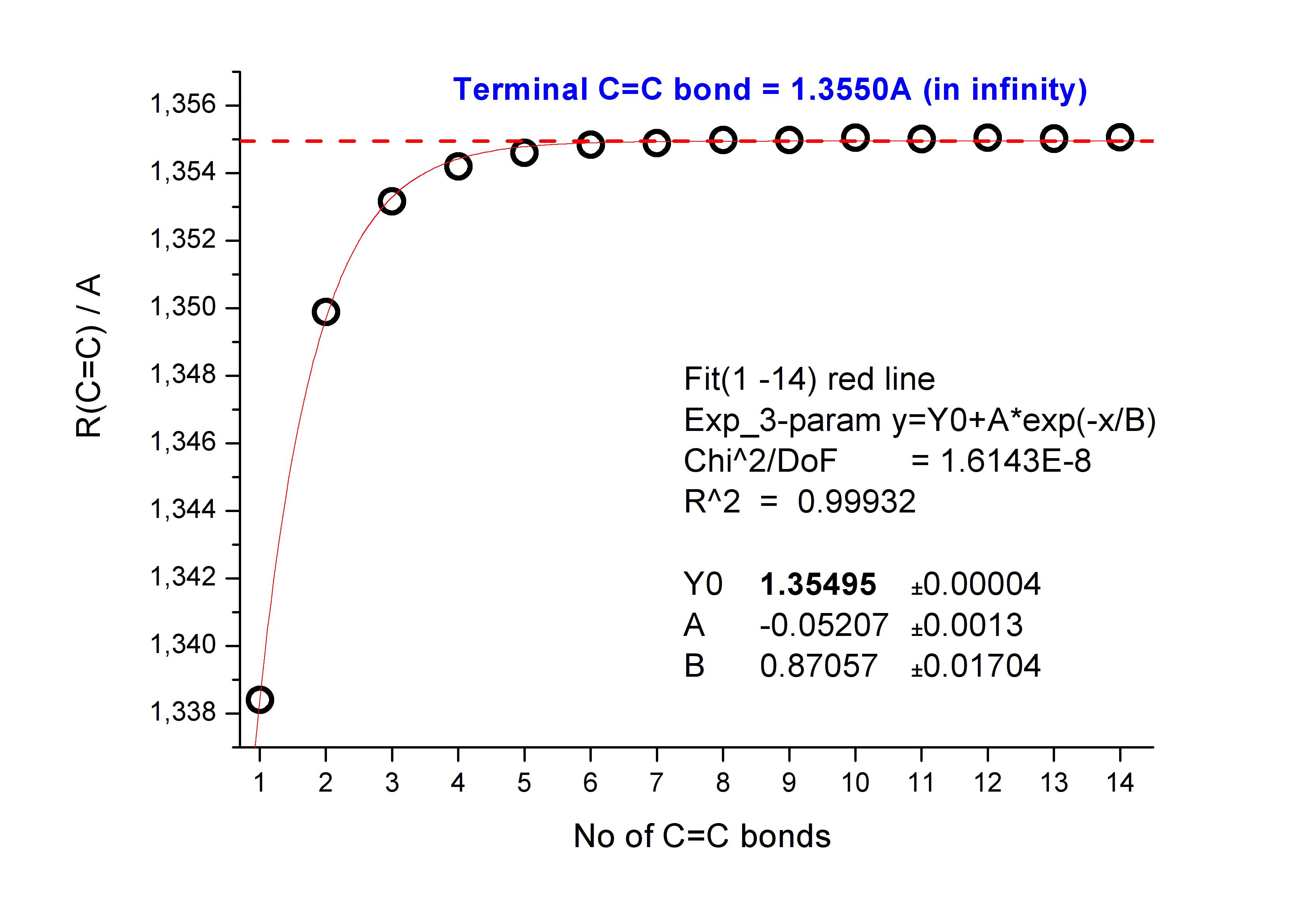
Figure S5B. Convergence of BLYP/6-311++G** calculated C=C bond length in the middle of all-cis polyene chains with 1 to 14 conjugated double bond units. The result of three-parameter fits using 1-14 data points is shown (red curve)


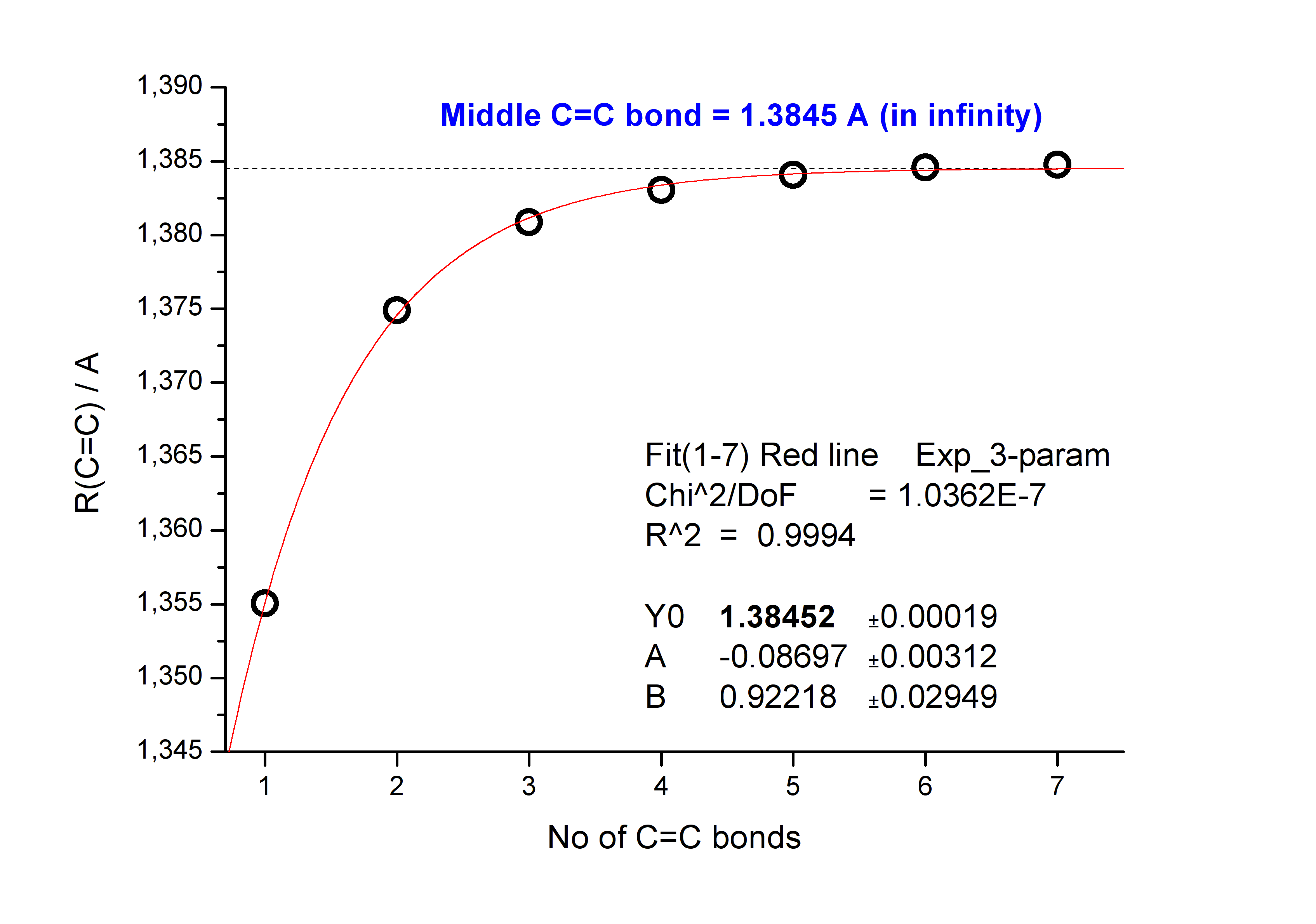
Figure S5C. Convergence of BLYP/6-311++G** calculated C=C bond length in the middle of all-cis polyene chains with 1 to 14 conjugated double bond units. The result of three-parameter fits using 1-7 data points is shown (red continuous line)


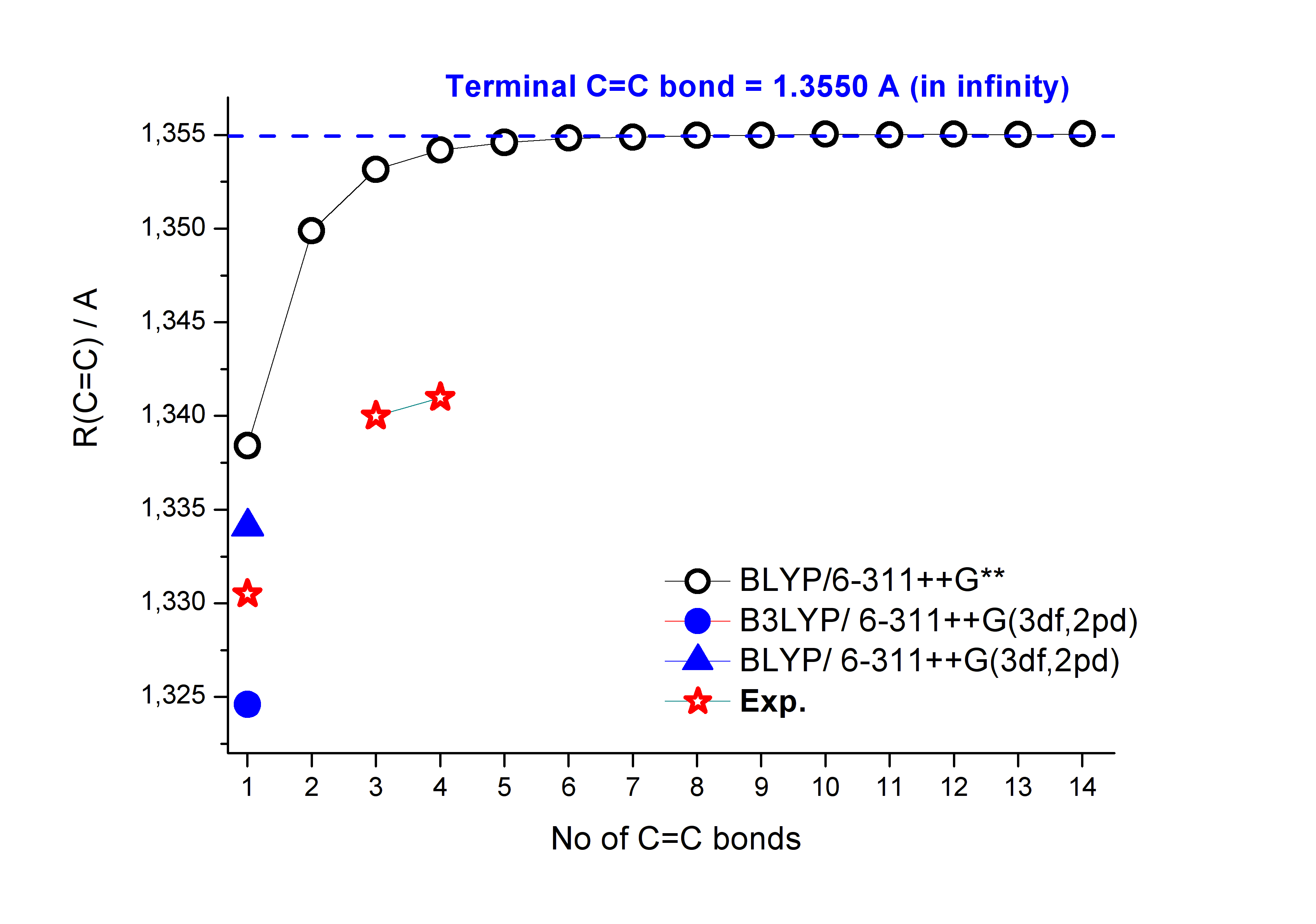
Figure S5D. Convergence of BLYP/6-311++G** calculated C=C bond length in the middle of all-cis polyene chains with 1 to 14 conjugated double bond units. Available experimental and benchmark theoretical results are also shown


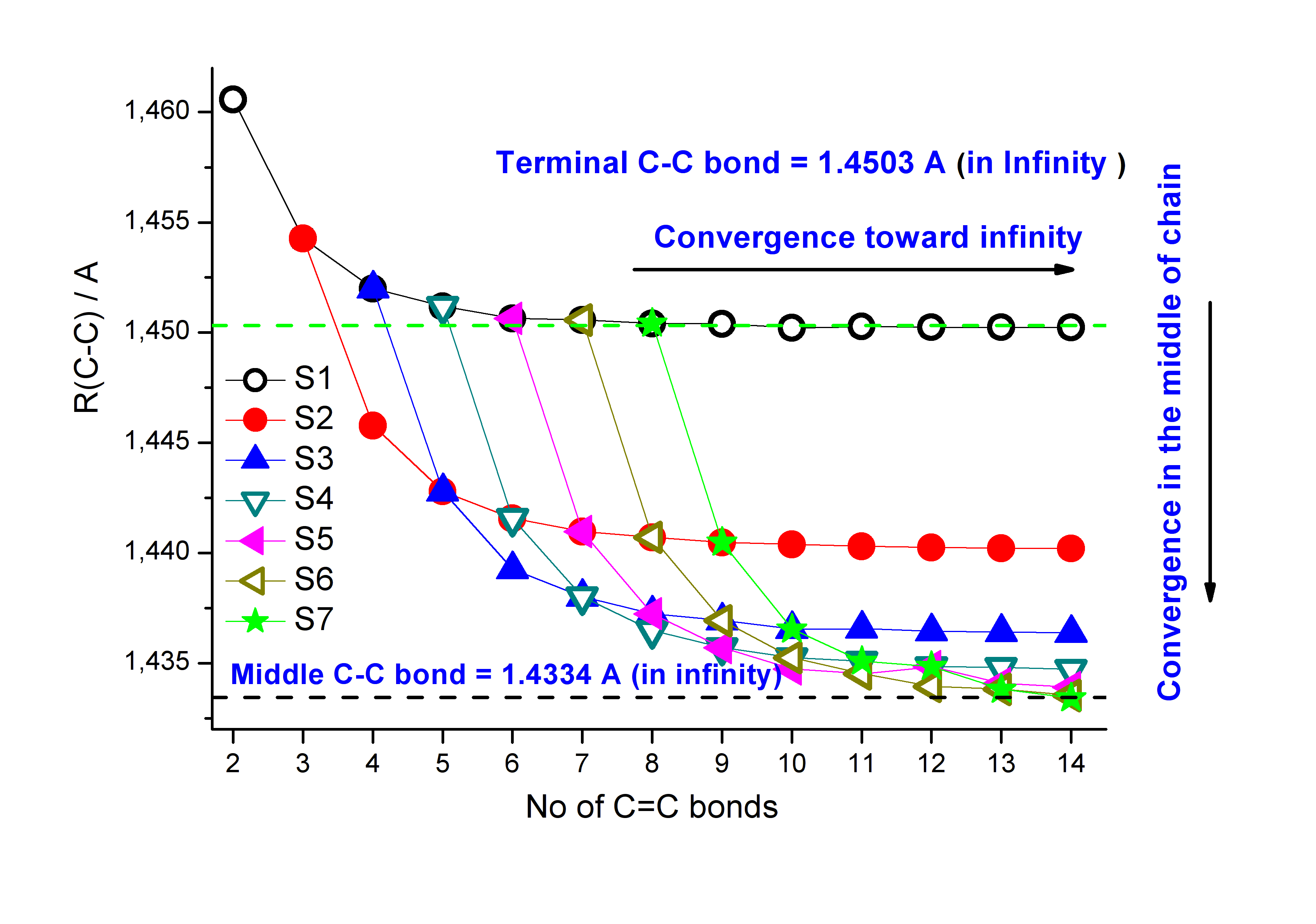
Figure S5E. Convergence of BLYP/6-311++G** calculated C-C bond length in all-cis polyene chains with 1 to 14 conjugated double bond units


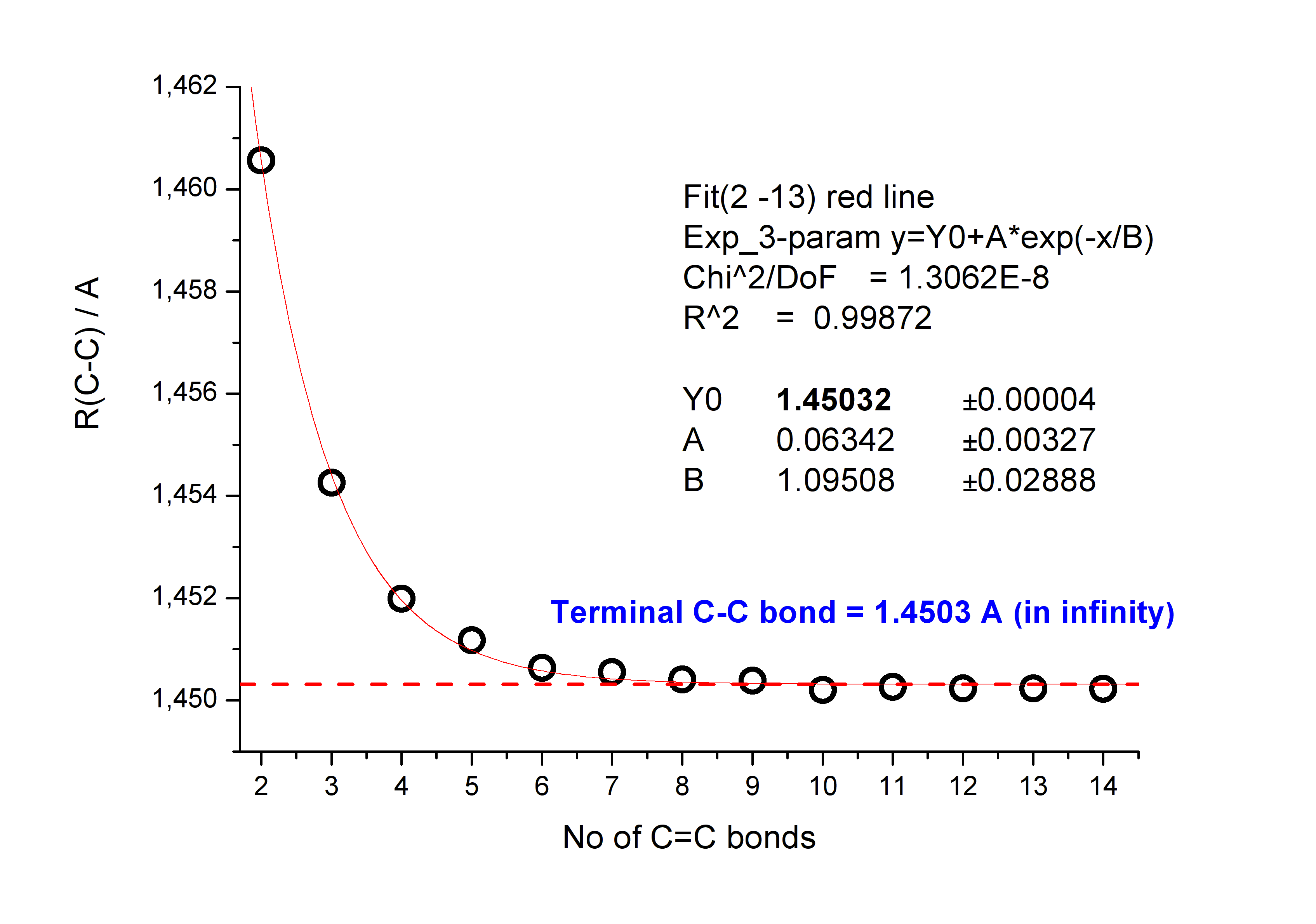
Figure S5F. Convergence of BLYP/6-311++G** calculated C-C bond length in all-cis polyene chains with 1 to 14 conjugated double bond units. The result of three-parameter fits using 1-14 data points is shown (red continuous line)


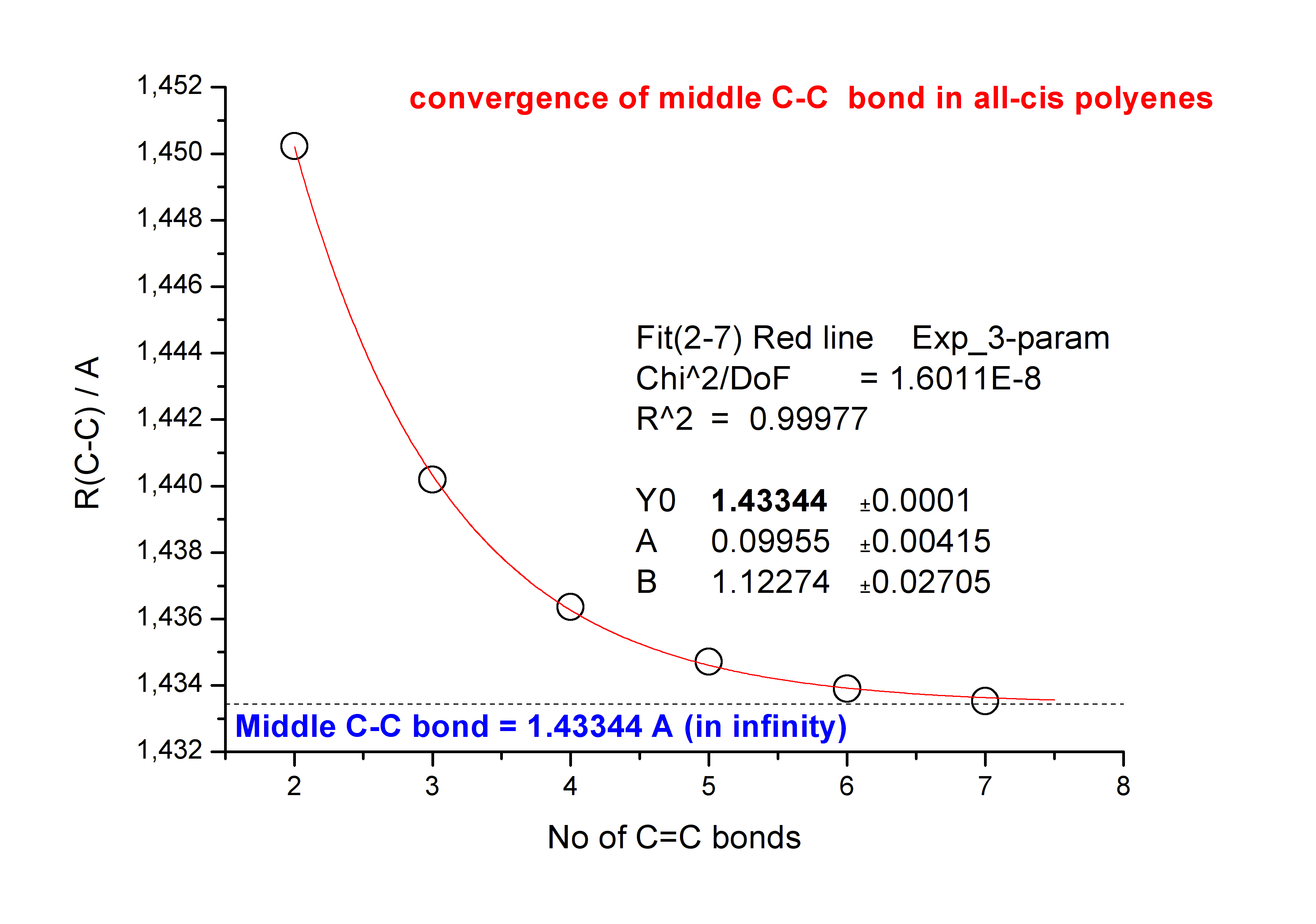
Figure S5G. Convergence of BLYP/6-311++G** calculated C-C bond length in all-cis polyene chains with 1 to 14 conjugated double bond units. The result of three-parameter fits using 1-6 data points is shown (red curve)


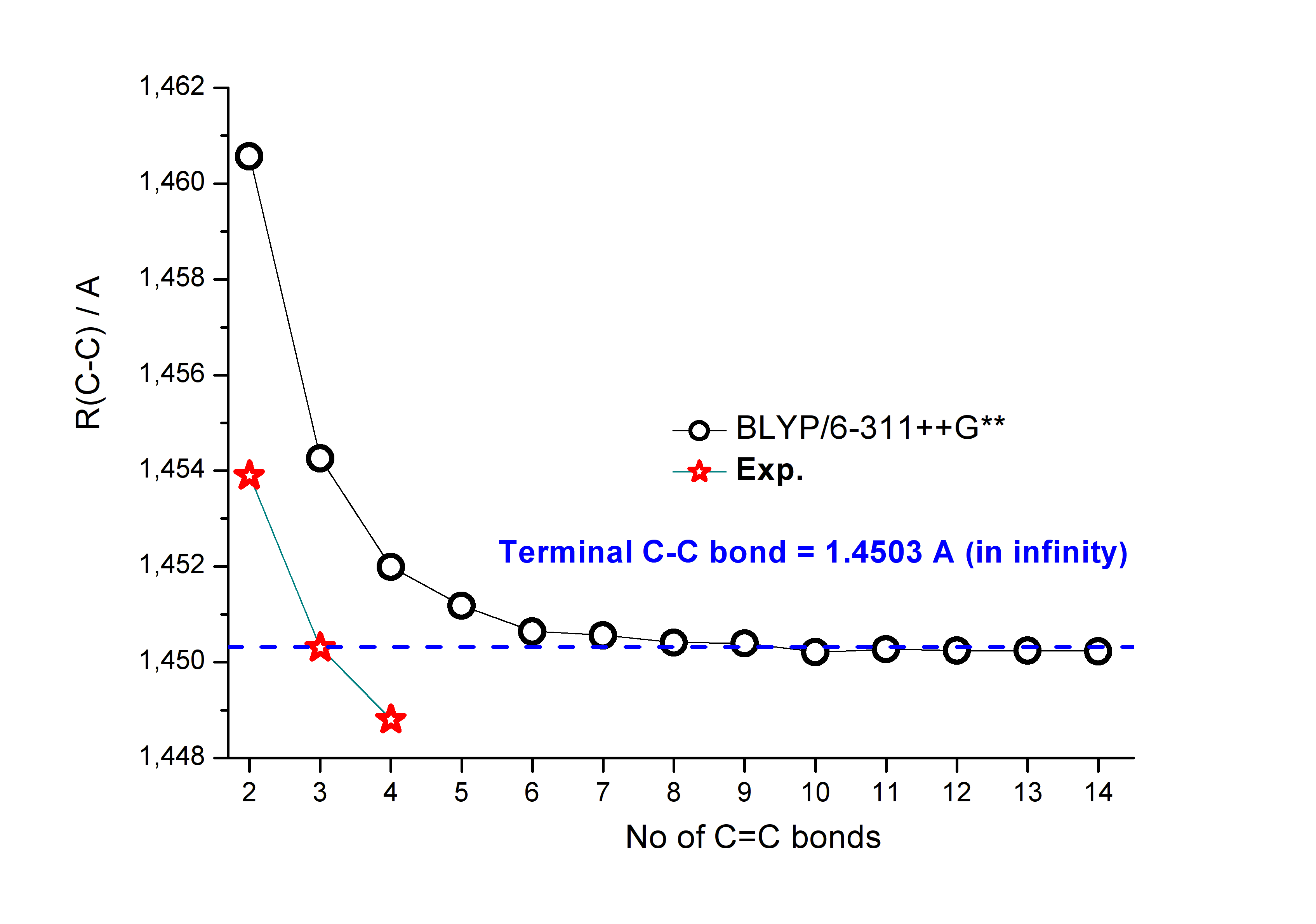
Figure S5H. Convergence of BLYP/6-311++G** calculated C-C bond length in all-cis polyene chains with 1 to 14 conjugated double bond units. Available experimental and benchmark theoretical results are also shown

**Table S1A.** B3LYP/6-311++G** calculated C=C bonds in ethylene and all-trans polyenes C2 – C14. To show subtle changes these data are reported with five decimal digits.

|  | D1 | D2 | D3 | D4 | D5 | D6 | D7 | D8 | D9 | D10 | D11 | D12 | D13 | D14 |
| --- | --- | --- | --- | --- | --- | --- | --- | --- | --- | --- | --- | --- | --- | --- |
| C1 | 1.32885 |  |  |  |  |  |  |  |  |  |  |  |  |  |
| C2 | 1.33834 | 1.33834 |  |  |  |  |  |  |  |  |  |  |  |  |
| C3 | 1.34097 | 1.34966 | 1.34097 |  |  |  |  |  |  |  |  |  |  |  |
| C4 | 1.34202 | 1.35315 | 1.35315 | 1.34202 |  |  |  |  |  |  |  |  |  |  |
| C5 | 1.34251 | 1.35462 | 1.35708 | 1.35462 | 1.34251 |  |  |  |  |  |  |  |  |  |
| C6 | 1.34277 | 1.35537 | 1.35881 | 1.35881 | 1.35537 | 1.34277 |  |  |  |  |  |  |  |  |
| C7 | 1.34293 | 1.35577 | 1.35973 | 1.36072 | 1.35973 | 1.35577 | 1.34293 |  |  |  |  |  |  |  |
| C8 | 1.34302 | 1.35602 | 1.36025 | 1.36175 | 1.36175 | 1.36025 | 1.35602 | 1.34302 |  |  |  |  |  |  |
| C9 | 1.34308 | 1.35618 | 1.36056 | 1.36234 | 1.36284 | 1.36234 | 1.36056 | 1.35618 | 1.34308 |  |  |  |  |  |
| C10 | 1.34312 | 1.35627 | 1.36076 | 1.36270 | 1.36348 | 1.36348 | 1.36270 | 1.36076 | 1.35627 | 1.34312 |  |  |  |  |
| C11 | 1.34314 | 1.35634 | 1.36089 | 1.36294 | 1.36388 | 1.36416 | 1.36388 | 1.36294 | 1.36089 | 1.35634 | 1.34314 |  |  |  |
| C12 | 1.34316 | 1.35638 | 1.36098 | 1.36309 | 1.36414 | 1.36459 | 1.36459 | 1.36414 | 1.36309 | 1.36098 | 1.35638 | 1.34316 |  |  |
| C13 | 1.34317 | 1.35641 | 1.36103 | 1.36319 | 1.36431 | 1.36486 | 1.36503 | 1.36486 | 1.36431 | 1.36319 | 1.36103 | 1.35641 | 1.34317 |  |
| C14 | 1.34318 | 1.35643 | 1.36107 | 1.36326 | 1.36443 | 1.36505 | 1.36532 | 1.36532 | 1.36505 | 1.36443 | 1.36326 | 1.36107 | 1.35643 | 1.34318 |

**Table S1B.** B3LYP/6-311++G** calculated C-C bonds in all-trans polyenes C2 – C14.

|  | S1 | S2 | S3 | S4 | S5 | S6 | S7 | S8 | S9 | S10 | S11 | S12 | S13 |
| --- | --- | --- | --- | --- | --- | --- | --- | --- | --- | --- | --- | --- | --- |
| C1 |  |  |  |  |  |  |  |  |  |  |  |  |  |
| C2 | 1.4562 |  |  |  |  |  |  |  |  |  |  |  |  |
| C3 | 1.44875 | 1.44875 |  |  |  |  |  |  |  |  |  |  |  |
| C4 | 1.44648 | 1.43984 | 1.44648 |  |  |  |  |  |  |  |  |  |  |
| C5 | 1.4455 | 1.43688 | 1.43688 | 1.4455 |  |  |  |  |  |  |  |  |  |
| C6 | 1.44499 | 1.43551 | 1.43353 | 1.43551 | 1.44499 |  |  |  |  |  |  |  |  |
| C7 | 1.44471 | 1.43478 | 1.43191 | 1.43191 | 1.43478 | 1.44471 |  |  |  |  |  |  |  |
| C8 | 1.44454 | 1.43435 | 1.43104 | 1.43015 | 1.43104 | 1.43435 | 1.44454 |  |  |  |  |  |  |
| C9 | 1.44443 | 1.4341 | 1.43051 | 1.42917 | 1.42917 | 1.43051 | 1.4341 | 1.44443 |  |  |  |  |  |
| C10 | 1.44436 | 1.43393 | 1.43019 | 1.42858 | 1.42812 | 1.42858 | 1.43019 | 1.43393 | 1.44436 |  |  |  |  |
| C11 | 1.44432 | 1.43382 | 1.42998 | 1.42821 | 1.42748 | 1.42748 | 1.42821 | 1.42998 | 1.43382 | 1.44432 |  |  |  |
| C12 | 1.44429 | 1.43375 | 1.42984 | 1.42797 | 1.42709 | 1.42682 | 1.42709 | 1.42797 | 1.42984 | 1.43375 | 1.44429 |  |  |
| C13 | 1.44426 | 1.4337 | 1.42975 | 1.42781 | 1.42682 | 1.42639 | 1.42639 | 1.42682 | 1.42781 | 1.42975 | 1.4337 | 1.44426 |  |
| C14 | 1.44425 | 1.43367 | 1.42969 | 1.42770 | 1.42665 | 1.42611 | 1.42595 | 1.42611 | 1.42665 | 1.42770 | 1.42969 | 1.43367 | 1.44425 |

**Table S1C.** B3LYP/6-311++G** calculated C=C bonds in ethylene and all-cis polyenes C2 – C14.

|  | D1 | D2 | D3 | D4 | D5 | D6 | D7 | D8 | D9 | D10 | D11 | D12 | D13 | D14 |
| --- | --- | --- | --- | --- | --- | --- | --- | --- | --- | --- | --- | --- | --- | --- |
| C1 | 1.32885 |  |  |  |  |  |  |  |  |  |  |  |  |  |
| C2 | 1.33834 | 1.33834 |  |  |  |  |  |  |  |  |  |  |  |  |
| C3 | 1.34097 | 1.35264 | 1.34097 |  |  |  |  |  |  |  |  |  |  |  |
| C4 | 1.3417 | 1.35661 | 1.35661 | 1.3417 |  |  |  |  |  |  |  |  |  |  |
| C5 | 1.34195 | 1.35772 | 1.36083 | 1.35772 | 1.34195 |  |  |  |  |  |  |  |  |  |
| C6 | 1.34209 | 1.35818 | 1.36216 | 1.36216 | 1.35818 | 1.34209 |  |  |  |  |  |  |  |  |
| C7 | 1.3421 | 1.35833 | 1.36264 | 1.36352 | 1.36264 | 1.35833 | 1.3421 |  |  |  |  |  |  |  |
| C8 | 1.34215 | 1.3584 | 1.36289 | 1.36409 | 1.36409 | 1.36289 | 1.3584 | 1.34215 |  |  |  |  |  |  |
| C9 | 1.34213 | 1.35846 | 1.36298 | 1.36434 | 1.36467 | 1.36434 | 1.36298 | 1.35846 | 1.34213 |  |  |  |  |  |
| C10 | 1.34217 | 1.35851 | 1.36307 | 1.3645 | 1.36498 | 1.36498 | 1.3645 | 1.36307 | 1.35851 | 1.34217 |  |  |  |  |
| C11 | 1.34214 | 1.35849 | 1.36306 | 1.36451 | 1.36507 | 1.36521 | 1.36507 | 1.36451 | 1.36306 | 1.35849 | 1.34214 |  |  |  |
| C12 | 1.34214 | 1.35849 | 1.36308 | 1.36454 | 1.36513 | 1.36534 | 1.36534 | 1.36513 | 1.36454 | 1.36308 | 1.35849 | 1.34214 |  |  |
| C13 | 1.34214 | 1.35849 | 1.36308 | 1.36455 | 1.36514 | 1.36536 | 1.36543 | 1.36536 | 1.36514 | 1.36455 | 1.36308 | 1.35849 | 1.34214 |  |
| C14 | 1.34214 | 1.35849 | 1.36309 | 1.36456 | 1.36516 | 1.3654 | 1.3655 | 1.3655 | 1.3654 | 1.36516 | 1.36456 | 1.36309 | 1.35849 | 1.34214 |

**Table S1D.** B3LYP/6-311++G** calculated C-C bonds in all-cis polyenes C2 – C14.

|  | S1 | S2 | S3 | S4 | S5 | S6 | S7 | S8 | S9 | S10 | S11 | S12 | S13 |
| --- | --- | --- | --- | --- | --- | --- | --- | --- | --- | --- | --- | --- | --- |
| C1 |  |  |  |  |  |  |  |  |  |  |  |  |  |
| C2 | 1.4562 |  |  |  |  |  |  |  |  |  |  |  |  |
| C3 | 1.45111 | 1.45111 |  |  |  |  |  |  |  |  |  |  |  |
| C4 | 1.44934 | 1.44413 | 1.44934 |  |  |  |  |  |  |  |  |  |  |
| C5 | 1.44877 | 1.4418 | 1.4418 | 1.44877 |  |  |  |  |  |  |  |  |  |
| C6 | 1.44841 | 1.44095 | 1.43906 | 1.44095 | 1.44841 |  |  |  |  |  |  |  |  |
| C7 | 1.44839 | 1.44055 | 1.4382 | 1.4382 | 1.44055 | 1.44839 |  |  |  |  |  |  |  |
| C8 | 1.4483 | 1.44041 | 1.43772 | 1.43714 | 1.43772 | 1.44041 | 1.4483 |  |  |  |  |  |  |
| C9 | 1.44831 | 1.44029 | 1.43756 | 1.43668 | 1.43668 | 1.43756 | 1.44029 | 1.44831 |  |  |  |  |  |
| C10 | 1.4482 | 1.44028 | 1.43733 | 1.43644 | 1.43607 | 1.43644 | 1.43733 | 1.44028 | 1.4482 |  |  |  |  |
| C11 | 1.44829 | 1.44023 | 1.43741 | 1.43635 | 1.43596 | 1.43596 | 1.43635 | 1.43741 | 1.44023 | 1.44829 |  |  |  |
| C12 | 1.44828 | 1.44023 | 1.43738 | 1.4363 | 1.43585 | 1.43573 | 1.43585 | 1.4363 | 1.43738 | 1.44023 | 1.44828 |  |  |
| C13 | 1.44824 | 1.44018 | 1.43734 | 1.43627 | 1.43583 | 1.43567 | 1.43567 | 1.43583 | 1.43627 | 1.43734 | 1.44018 | 1.44824 |  |
| C14 | 1.44824 | 1.44017 | 1.43732 | 1.43624 | 1.43579 | 1.4356 | 1.43556 | 1.4356 | 1.43579 | 1.43624 | 1.43732 | 1.44017 | 1.44824 |

**Table S2A.** BLYP/6-311++G** calculated C=C bonds in ethylene and all-trans polyenes C2 – C14.

|  | D1 | D2 | D3 | D4 | D5 | D6 | D7 | D8 | D9 | D10 | D11 | D12 | D13 | D14 |
| --- | --- | --- | --- | --- | --- | --- | --- | --- | --- | --- | --- | --- | --- | --- |
| C1 | 1.33841 |  |  |  |  |  |  |  |  |  |  |  |  |  |
| C2 | 1.34989 | 1.34989 |  |  |  |  |  |  |  |  |  |  |  |  |
| C3 | 1.35336 | 1.36368 | 1.35336 |  |  |  |  |  |  |  |  |  |  |  |
| C4 | 1.3548 | 1.36811 | 1.36811 | 1.3548 |  |  |  |  |  |  |  |  |  |  |
| C5 | 1.35553 | 1.37015 | 1.37318 | 1.37015 | 1.35553 |  |  |  |  |  |  |  |  |  |
| C6 | 1.35595 | 1.37129 | 1.37562 | 1.37562 | 1.37129 | 1.35595 |  |  |  |  |  |  |  |  |
| C7 | 1.35623 | 1.37196 | 1.37698 | 1.37828 | 1.37698 | 1.37196 | 1.35623 |  |  |  |  |  |  |  |
| C8 | 1.3564 | 1.37241 | 1.37784 | 1.37982 | 1.37982 | 1.37784 | 1.37241 | 1.3564 |  |  |  |  |  |  |
| C9 | 1.35653 | 1.37273 | 1.37843 | 1.38083 | 1.38151 | 1.38083 | 1.37843 | 1.37273 | 1.35653 |  |  |  |  |  |
| C10 | 1.35663 | 1.37295 | 1.37884 | 1.38150 | 1.38260 | 1.38260 | 1.38150 | 1.37884 | 1.37295 | 1.35663 |  |  |  |  |
| C11 | 1.35670 | 1.37312 | 1.37913 | 1.38198 | 1.38334 | 1.38376 | 1.38334 | 1.38198 | 1.37913 | 1.37311 | 1.35670 |  |  |  |
| C12 | 1.35675 | 1.37324 | 1.37935 | 1.38233 | 1.38388 | 1.38456 | 1.38456 | 1.38388 | 1.38233 | 1.37935 | 1.37324 | 1.35675 |  |  |
| C13 | 1.35679 | 1.37333 | 1.37952 | 1.3826 | 1.38428 | 1.38514 | 1.38541 | 1.38514 | 1.38428 | 1.3826 | 1.37952 | 1.37333 | 1.35679 |  |
| C14 | 1.35682 | 1.37341 | 1.37965 | 1.3828 | 1.38459 | 1.38558 | 1.38603 | 1.38603 | 1.38558 | 1.38459 | 1.3828 | 1.37965 | 1.37341 | 1.35682 |

**Table S2B.** BLYP/6-311++G** calculated C-C bonds in all-trans polyenes C2 – C14.

|  | S1 | S2 | S3 | S4 | S5 | S6 | S7 | S8 | S9 | S10 | S11 | S12 | S13 |
| --- | --- | --- | --- | --- | --- | --- | --- | --- | --- | --- | --- | --- | --- |
| C1 |  |  |  |  |  |  |  |  |  |  |  |  |  |
| C2 | 1.46057 |  |  |  |  |  |  |  |  |  |  |  |  |
| C3 | 1.45178 | 1.45178 |  |  |  |  |  |  |  |  |  |  |  |
| C4 | 1.44891 | 1.44133 | 1.44891 |  |  |  |  |  |  |  |  |  |  |
| C5 | 1.44757 | 1.43761 | 1.43761 | 1.44757 |  |  |  |  |  |  |  |  |  |
| C6 | 1.44686 | 1.43577 | 1.4334 | 1.43577 | 1.44686 |  |  |  |  |  |  |  |  |
| C7 | 1.44637 | 1.4347 | 1.43121 | 1.43121 | 1.4347 | 1.44637 |  |  |  |  |  |  |  |
| C8 | 1.44609 | 1.43402 | 1.42993 | 1.42882 | 1.42993 | 1.43402 | 1.44609 |  |  |  |  |  |  |
| C9 | 1.44589 | 1.43357 | 1.42909 | 1.42737 | 1.42737 | 1.42909 | 1.43357 | 1.44589 |  |  |  |  |  |
| C10 | 1.44574 | 1.43324 | 1.42852 | 1.42642 | 1.42581 | 1.42642 | 1.42852 | 1.43324 | 1.44574 |  |  |  |  |
| C11 | 1.44563 | 1.43300 | 1.42810 | 1.42576 | 1.42477 | 1.42477 | 1.42576 | 1.42810 | 1.43300 | 1.44563 |  |  |  |
| C12 | 1.44555 | 1.43283 | 1.4278 | 1.42528 | 1.42404 | 1.42366 | 1.42404 | 1.42528 | 1.4278 | 1.43283 | 1.44555 |  |  |
| C13 | 1.44548 | 1.43269 | 1.42757 | 1.42492 | 1.42351 | 1.42288 | 1.42288 | 1.42351 | 1.42492 | 1.42757 | 1.43269 | 1.44548 |  |
| C14 | 1.44543 | 1.43259 | 1.42739 | 1.42465 | 1.42311 | 1.42231 | 1.42206 | 1.42231 | 1.42311 | 1.42465 | 1.42739 | 1.43259 | 1.44543 |

**Table S2C.** BLYP/6-311++G** calculated C=C bonds in ethylene and all-cis polyenes C2 – C14.

|  | D1 | D2 | D3 | D4 | D5 | D6 | D7 | D8 | D9 | D10 | D11 | D12 | D13 | D14 |
| --- | --- | --- | --- | --- | --- | --- | --- | --- | --- | --- | --- | --- | --- | --- |
| C1 | 1.33841 |  |  |  |  |  |  |  |  |  |  |  |  |  |
| C2 | 1.34989 | 1.34989 |  |  |  |  |  |  |  |  |  |  |  |  |
| C3 | 1.35317 | 1.36715 | 1.35317 |  |  |  |  |  |  |  |  |  |  |  |
| C4 | 1.3542 | 1.37186 | 1.37186 | 1.3542 |  |  |  |  |  |  |  |  |  |  |
| C5 | 1.3546 | 1.37341 | 1.37717 | 1.37341 | 1.3546 |  |  |  |  |  |  |  |  |  |
| C6 | 1.35484 | 1.37414 | 1.37905 | 1.37905 | 1.37414 | 1.35484 |  |  |  |  |  |  |  |  |
| C7 | 1.35489 | 1.37443 | 1.37984 | 1.38100 | 1.37984 | 1.37443 | 1.35489 |  |  |  |  |  |  |  |
| C8 | 1.35498 | 1.37458 | 1.38028 | 1.38193 | 1.38193 | 1.38028 | 1.37458 | 1.35498 |  |  |  |  |  |  |
| C9 | 1.35498 | 1.37471 | 1.38049 | 1.38239 | 1.38289 | 1.38239 | 1.38049 | 1.37471 | 1.35498 |  |  |  |  |  |
| C10 | 1.35505 | 1.37481 | 1.38068 | 1.38271 | 1.38346 | 1.38346 | 1.38271 | 1.38068 | 1.37481 | 1.35505 |  |  |  |  |
| C11 | 1.35501 | 1.37479 | 1.38071 | 1.38278 | 1.38365 | 1.38387 | 1.38365 | 1.38278 | 1.38071 | 1.37479 | 1.35501 |  |  |  |
| C12 | 1.35505 | 1.37487 | 1.38081 | 1.38297 | 1.38391 | 1.38429 | 1.38429 | 1.38391 | 1.38297 | 1.38081 | 1.37487 | 1.35505 |  |  |
| C13 | 1.35502 | 1.37483 | 1.3808 | 1.38295 | 1.38393 | 1.38434 | 1.38448 | 1.38434 | 1.38393 | 1.38295 | 1.3808 | 1.37483 | 1.35502 |  |
| C14 | 1.35506 | 1.37489 | 1.38086 | 1.38305 | 1.38407 | 1.38456 | 1.38476 | 1.38476 | 1.38456 | 1.38407 | 1.38305 | 1.38086 | 1.37489 | 1.35506 |

**Table S2D.** BLYP/6-311++G** calculated C-C bonds in all-cis polyenes C2 – C14.

|  | S1 | S2 | S3 | S4 | S5 | S6 | S7 | S8 | S9 | S10 | S11 | S12 | S13 |
| --- | --- | --- | --- | --- | --- | --- | --- | --- | --- | --- | --- | --- | --- |
| C1 |  |  |  |  |  |  |  |  |  |  |  |  |  |
| C2 | 1.46057 |  |  |  |  |  |  |  |  |  |  |  |  |
| C3 | 1.45426 | 1.45426 |  |  |  |  |  |  |  |  |  |  |  |
| C4 | 1.45199 | 1.44578 | 1.45199 |  |  |  |  |  |  |  |  |  |  |
| C5 | 1.45118 | 1.44279 | 1.44279 | 1.45118 |  |  |  |  |  |  |  |  |  |
| C6 | 1.45064 | 1.44156 | 1.43925 | 1.44156 | 1.45064 |  |  |  |  |  |  |  |  |
| C7 | 1.45056 | 1.44097 | 1.43800 | 1.43800 | 1.44097 | 1.45056 |  |  |  |  |  |  |  |
| C8 | 1.45041 | 1.44071 | 1.43724 | 1.43647 | 1.43724 | 1.44071 | 1.45041 |  |  |  |  |  |  |
| C9 | 1.45039 | 1.44048 | 1.43692 | 1.4357 | 1.4357 | 1.43692 | 1.44048 | 1.45039 |  |  |  |  |  |
| C10 | 1.45021 | 1.44039 | 1.43654 | 1.43525 | 1.43473 | 1.43525 | 1.43654 | 1.44039 | 1.45021 |  |  |  |  |
| C11 | 1.45027 | 1.44029 | 1.43656 | 1.43508 | 1.43451 | 1.43451 | 1.43508 | 1.43656 | 1.44029 | 1.45027 |  |  |  |
| C12 | 1.45024 | 1.44024 | 1.43644 | 1.43484 | 1.43484 | 1.43394 | 1.43484 | 1.43484 | 1.43644 | 1.44024 | 1.45024 |  |  |
| C13 | 1.45024 | 1.44021 | 1.43641 | 1.43481 | 1.43408 | 1.43381 | 1.43381 | 1.43408 | 1.43481 | 1.43641 | 1.44021 | 1.45024 |  |
| C14 | 1.45023 | 1.4402 | 1.43636 | 1.43471 | 1.4339 | 1.43353 | 1.43341 | 1.43353 | 1.4339 | 1.43471 | 1.43636 | 1.4402 | 1.45023 |

**Table S3.** Deviations^a^ of BLYP/6-311++G** calculated ν_(C=C)_ and ν_(C-C)_ in C3 to C12 all-trans polyenes (in cm^-1^)

with ends capped with t-butyl groups from experiment^b^.

| ***n*** | **ν_(C=C)_** | | | **ν_(C-C)_** | | |
| --- | --- | --- | --- | --- | --- | --- |
|  | **Calc.** | **Exp.** | **Δ** | **Calc.** | **Exp.** | **Δ** |
| 3 | 1605.1 | 1638.5 | -33.4 | 1121.49 | 1136.1 | -14.61 |
| 4 | 1579.84 | 1613.8 | -33.96 | 1130.42 | 1142.5 | -12.08 |
| 5 | 1556.76 | 1587.3 | -30.54 | 1137.43 | 1145.4 | -7.97 |
| 6 | 1536.93 | 1570.6 | -33.67 | 1140.85 | 1144.5 | -3.65 |
| 7 | 1520.03 | 1555.6 | -35.57 | 1141.66 | 1143.7 | -2.04 |
| 8 | 1505.57 | 1542.2 | -36.63 | 1140.93 | 1140.1 | 0.83 |
| 9 | 1493.18 | 1530.9 | -37.72 | 1137.02 | 1134.9 | 2.12 |
| 10 | 1482.7 | 1520.9 | -38.2 | 1126.96 | 1129.8 | -2.84 |
| 11 | 1473.05 | 1514 | -40.95 | 1117.48 | 1125.1 | -7.62 |
| 12 | 1465.58 | 1505.9 | -40.32 | 1112.42 | 1121.8 | -9.38 |

a) Δ = Calc. – Exp.; b) from ref. [3]

**Table S4.** BLYP/6-311++G** calculated and corrected ν_(str. C=C)_ and ν_(str. C-C)_

frequencies of ethylene and C2 – C14 all-trans polyenes (in cm^-1^)

| **C_n_** | **ν_(C=C)_** | | **ν_(C-C)_** | |
| --- | --- | --- | --- | --- |
|  | **raw** | **corrected** | **raw** | **corrected** |
| 1 | 1628.93 | 1656.88 |  |  |
| 2 | 1629.93 | 1659.05 | 1193.86 | 1210.7 |
| 3 | 1607.24 | 1637.52 | 1185.64 | 1199.58 |
| 4 | 1582.56 | 1614.00 | 1180.06 | 1191.1 |
| 5 | 1556.76 | 1592.03 | 1177.60 | 1180.99 |
| 6 | 1538.25 | 1572.02 | 1164.46 | 1169.7 |
| 7 | 1521.86 | 1556.79 | 1158.78 | 1161.12 |
| 8 | 1506.94 | 1543.03 | 1152.37 | 1151.81 |
| 9 | 1488.67 | 1525.93 | 1131.3 | 1127.84 |
| 10 | 1478.27 | 1516.69 | 1123.23 | 1125.7 |
| 11 | 1469.2 | 1508.78 | 1116.21 | 1122.6 |
| 12 | 1465.43 | 1506.18 | 1113.52 | 1123.84 |
| 13 | 1456.16 | 1498.07 | 1105.66 | 1119.91 |
| 14 | 1451.75 | 1494.82 | 1097.77 | 1115.95 |
| **Red coral** | **1520** | | **1131** | |
